# Reduced chromatin accessibility correlates with resistance to Notch activation

**DOI:** 10.1038/s41467-022-29834-z

**Published:** 2022-04-25

**Authors:** Jelle van den Ameele, Robert Krautz, Seth W. Cheetham, Alex P. A. Donovan, Oriol Llorà-Batlle, Rebecca Yakob, Andrea H. Brand

**Affiliations:** 1 The Gurdon Institute and Department of Physiology, Development and Neuroscience, University of Cambridge, Cambridge, UK; 2 Present Address: MRC Mitochondrial Biology Unit and Department of Clinical Neurosciences, University of Cambridge, Cambridge, UK; 3 Present Address: The Bioinformatics Centre, Department of Biology, University of Copenhagen, Copenhagen, Denmark; 4 Present Address: Mater Research Institute-University of Queensland, Woolloongabba, Australia

**Keywords:** Differentiation, Neurogenesis

## Abstract

The Notch signalling pathway is a master regulator of cell fate transitions in development and disease. In the brain, Notch promotes neural stem cell (NSC) proliferation, regulates neuronal migration and maturation and can act as an oncogene or tumour suppressor. How NOTCH and its transcription factor RBPJ activate distinct gene regulatory networks in closely related cell types in vivo remains to be determined. Here we use Targeted DamID (TaDa), requiring only thousands of cells, to identify NOTCH and RBPJ binding in NSCs and their progeny in the mouse embryonic cerebral cortex in vivo. We find that NOTCH and RBPJ associate with a broad network of NSC genes. Repression of NSC-specific Notch target genes in intermediate progenitors and neurons correlates with decreased chromatin accessibility, suggesting that chromatin compaction may contribute to restricting NOTCH-mediated transactivation.

## Abstract (web summary)

Notch signalling plays a key role in cell fate transitions, but how Notch activates distinct regulatory networks in closely related cell types is not well understood. Here the authors profile Notch and RBPJ targets in the developing mouse cortex and reveal how transcription factor occupancy and chromatin remodelling interact to direct differential gene expression during differentiation.

## Introduction

**Fig. 1:**
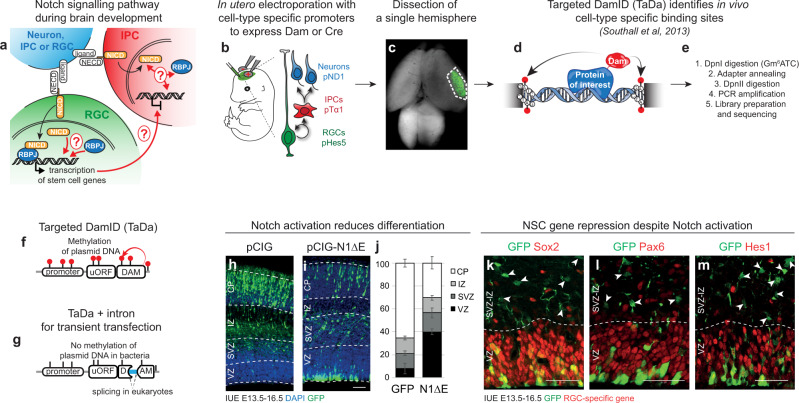
*In utero* cell-type specific chromatin profiling. **a** Overview of the Notch pathway in the developing cortex. **b**–**e** Workflow of *in utero* Targeted DamID (TaDa). **f**, **g** Schematic overview of TaDa construct and the introduction of an intron to prevent methylation in bacteria and enable transient transfection. **h**–**m**. IUE of **h** pCIG or **i** pCIG-N1ΔE labelled with GFP (green) and DAPI (blue) or **k**–**m** RGC-specific genes (*Sox2* (*n* = 1), *Pax6* (*n* = 3) or *Hes1* (*n* = 3), red). **j** Mean percentage (±s.e.m.) of GFP-positive cells in each region (*n* = 3 embryos (pCIG) and *n* = 5 embryos (pCIG-N1ΔE)). Source data are provided as a Source Data file. Scale bars 100 µm **h**, **i**, and 50 µm **k**–**m**.

Notch signalling plays a key role in cell fate transitions during the differentiation of neural lineages, from neural stem cells (NSCs) to postmitotic neurons^1^. Upon ligand binding, the NOTCH receptor is cleaved and the intracellular domain (NICD) translocates to the nucleus where it associates with its cofactor, RBPJ, to activate target gene expression^1,2^ (Fig. 1a). The outcome of Notch pathway signalling is highly dependent upon cellular context. In the developing mouse brain, Notch activity promotes the proliferation of radial glial cells (RGCs) and prevents their differentiation towards intermediate progenitor cells (IPCs) and neurons^3–7^. However, Notch also controls neuronal maturation^8–10^, promotes the proliferation of IPCs independently of RBPJ^4^ and, depending upon the cell of origin, sustained Notch activity in the brain can either drive or prevent tumourigenesis^11,12^. How a single signalling pathway can exert these pleiotropic effects during cell fate transitions, in closely related cell types, is a key question in developmental and cancer biology.

Here, we use Targeted DamID (TaDa)^13–18^ to perform cell-type specific genome-wide profiling of NOTCH and RBPJ binding in vivo in the developing mouse brain. This reveals dynamic binding patterns in two directly related cell types, RGCs and IPCs, and enables us to compare binding, chromatin accessibility and gene expression during differentiation.

## Results

### Targeted DamID for cell-type specific profiling in vivo during mammalian development

Downregulation of Notch target genes during neurogenesis has been proposed to result from differential chromatin binding of Notch and RBPJ in RGCs and IPCs^4^. However, despite their remarkable context-dependent function, cell-type specific binding profiles of NOTCH and RBPJ during mammalian nervous system development have not been determined. This is largely due to the difficulty of capturing NICD in the nucleus^19–21^, the short half-lives of both NICD and its canonical target genes^2,22^ and the dependence of Notch-signalling on cell-cell contact, which is disrupted by cell sorting. Insights into cell-type specificity of Notch pathway output have come from studying transcriptional regulation of NOTCH-target genes in model organisms^2,23–27^. A simple approach for assessing cell type specific Notch and RBPJ binding patterns, in vivo and genome-wide, would help to reveal how cell-type specific target gene expression is achieved.

Here we used TaDa after *in utero* electroporation (IUE)^18^ to map NOTCH and RBPJ binding in the developing mouse brain (Fig. 1b–e). TaDa was originated to enable cell type-specific profiling without cell isolation, allowing genome-wide profiling of DNA- or chromatin-binding proteins without cell sorting, fixation or affinity purification^13–16^. TaDa is extremely sensitive and highly reproducible in *Drosophila*, as well as in mammalian cells, starting from fewer than 10,000 cells^13–18,28^. Nonetheless, DamID and TaDa were considered incompatible with transient transfection: the Dam-fusion protein methylates plasmid DNA when expressed in bacteria^29^. The methylated plasmid DNA co-amplifies with genomic DNA, comprising a substantial proportion of the sequencing library^29^. To overcome this, an intron was introduced into the coding sequence of the Dam methylase to prevent plasmid methylation in bacteria without affecting methylation in eukaryotes (intronDam^18^; Fig. 1g; Supplementary Fig. 1a, b; see Methods). To test this, we expressed Dam methylase alone under the control of an ubiquitous promoter (CAG; mouse embryonic stage E13.5), which should reveal regions of accessible chromatin (CaTaDa^15,30^). IUE of intronDam-plasmids generated reproducible methylation patterns in single brain hemispheres at E17.5 (Supplementary Fig. 2a; Supplementary Fig. 3a**)** without seemingly affecting neurogenesis (DamID-seq^14,15^; Supplementary Fig. 2b–d). The *in utero* Dam-methylation peaks corresponded with previously published ATAC-seq peaks from E13.5 mouse forebrain^31,32^ (Supplementary Fig. 2e, f), consistent with preferential methylation of accessible chromatin by untethered Dam methylase^30,33^.

We designed a Cre-inducible construct for conditional expression and to prevent leaky expression (‘non-leaky’ floxDam). The N-terminal half of the Dam methylase sequence was inverted and flanked by loxP sites (Supplementary Fig. 4a). Cre induced recombination activates expression of both the primary open reading frame (uORF, mCherry) and, at far lower levels, the secondary ORF encoding the Dam methylase (Supplementary Fig. 4b–d).

### Notch pathway activation in RGCs is not sufficient to prevent neurogenesis entirely

Notch and RBPJ are key regulators of RGC proliferation and prevent the transition of RGCs into IPCs and postmitotic neurons^1^. Notch1 mRNA levels decrease significantly upon transition from RGCs to IPCs^34,35^ (Supplementary Fig. 5a): Notch1 is expressed at low levels in IPCs but not in neurons. We therefore tested whether neurogenesis could proceed if the Notch pathway were activated ectopically. Notch pathway activation by ectopic expression of NICD has been shown previously to upregulate RGC-specific genes involved in NSC maintenance^3,4,36,37^. As expected, ectopic expression of NICD in RGCs after *in utero* electroporation (IUE) led to a strong reduction in differentiation (Fig. 1h–j). However, neurogenesis was not entirely blocked: 30.5 ± 5.5% (*n* = 5 embryos) of cells were able to differentiate into postmitotic neurons of the cortical plate (CP) after 3 days (Fig. 1j). Expression of RGC-specific transcription factors, *Sox2* and *Pax6* (Fig. 1k, l), or the NOTCH-target gene *Hes1* (Fig. 1m), was undetectable in IPCs and neurons. Despite expression of GFP and NICD from the same bicistronic transcript, NICD may no longer be present in these GFP-labelled IPCs and neurons. However, if still present, NICD would appear to be insufficient to activate NSC target genes or to completely block lineage progression. NICD might be unable to bind to its target genes in IPCs or, if able to bind, transcription might be repressed by other means.

**Fig. 2:**
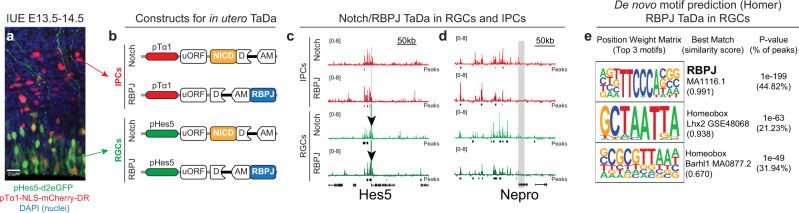
Cell-type specific *in utero* TaDa of NOTCH and RBPJ. **a** IUE of plasmids with destabilized fluorescent proteins under control of *Hes5* and *Tα1* promoters (n = 1). Staining for DAPI (nuclei, blue), GFP (green) and mCherry (red). Scale bar, 20 µm. **b** Constructs used for NOTCH and RBPJ TaDa in RGCs and IPCs. **c**, **d** NOTCH and RBPJ binding peaks near known target genes (grey shading) in RGCs and IPCs. Arrowheads indicate *Hes5*-promoter present in p*Hes5*-plasmids. **e** Motif detection at RBPJ binding peaks in RGCs.

To assess whether Notch could bind to chromatin in differentiating cells, we profiled NOTCH and RBPJ1 binding in the developing mouse cortex by TaDa. TaDa is highly sensitive, generating robust results from fewer than 10,000 cultured cells^17^ and should enable small populations of cells to be assayed in vivo. We performed IUE with plasmids expressing RBPJ1 and NOTCH1 (NICD or NΔECD; see Methods) fused to intronDam, under the control of RGC-specific (Hes5)^4,38^ or IPC-specific promoters (Tα1^4,39,40^; Fig. 2a, b; Supplementary Fig. 3a, b; Supplementary Fig. 5b).

At a stringent FDR < 10^−5^, we identified 14,071 peaks in RGC^RBPJ^; 3688 peaks in RGC^Notch^; 5454 peaks in IPC^RBPJ^; 2099 peaks in IPC^Notch^; (Supplementary Fig. 5c; Supplementary Data 1). As expected, genes near Notch/RBPJ peaks were highly enriched for gene ontology (GO) terms related to neurogenesis and the Notch pathway (Supplementary Fig. 5d–f) and included well-known Notch target genes, such as *Hes1*, *Hes5*^36^, *Nrarp*^41^ or *Nepro*^42^ (Fig. 2c, d). Of the 1910 genes previously found to be differentially expressed upon NICD expression in the cortex^37^, 888/1910 (46.5%) were associated with RGC^RBPJ^ peaks, as compared to 455/1910 (23.8%) found previously by RBPJ ChIP-seq of cortical NSCs^37^ (Supplementary Fig. 5g,h; Supplementary Data 2). We found many NSC genes that had not yet been identified as Notch targets (Supplementary Data 1), as well as previously identified targets, such as transcriptional effectors of the Wnt, FGF and Shh signalling pathways^37^ (Supplementary Fig. 5e, f), including *Ctnnb1*, *Tcf7l2 (Tcf4)* and *Gli2/3*, and many upstream genes involved in signal transduction (Supplementary Data 1).

The most highly enriched motif under the RGC^RBPJ^ peaks matches the known consensus binding site of RBPJ: 5′-TTCCCA-3’^43^ (*p* = 1^−199^; 44.82% of peaks; Fig. 2e; Supplementary Fig. 5i). In addition, consensus binding sites for homeobox transcription factors, in particular LHX2, were significantly enriched at Notch and RBPJ peaks in both RGCs and IPCs peaks (Fig. 2e; Supplementary Fig. 5j). LHX2 is necessary for the activation of several Notch target genes in the developing retina^44^ and our data suggest a similar requirement in cortical NSCs, possibly through increasing enhancer accessibility by establishing a permissive chromatin state^2,45^.

### Chromatin accessibility correlates with Notch and RBPJ binding

**Fig. 3:**
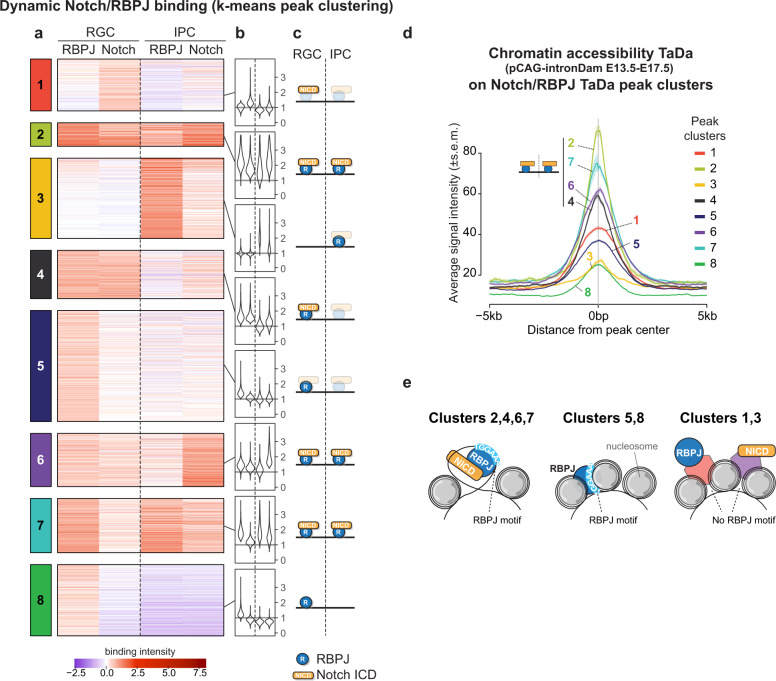
Dynamic NOTCH/RBPJ binding during neurogenesis. **a**–**c** k-means clustering of all regions bound by NOTCH or RBPJ in RGCs or IPCs results in 8 clusters corresponding to distinct binding patterns of RBPJ (blue) and NICD (yellow). Colour scale indicates TaDa binding intensity. **d** pCAG-iDam average signal (±s.e.m., shaded area) on NOTCH /RBPJ peak clusters. **e** Peak clusters indicate chromatin accessibility and binding mode.

Peaks from the four datasets (*n* = 20,811) were clustered using an optimisation approach based on k-means (Supplementary Fig. 6; Supplementary Data 3), revealing eight robust subsets of peaks (Fig. 3a, b). Consistent with the prevailing view of how *Notch* regulates target gene expression^46^, many binding sites were characterized by binding of both NOTCH and RBPJ in RGCs (cluster 4), or in both RGCs and IPCs (clusters 2, 6 and 7) (Fig. 3a–c). The majority of peaks, however, showed preferential binding of either NOTCH or RBPJ, but not both: NOTCH alone in RGCs (cluster 1), RBPJ alone in RGCs (clusters 5 and 8), or RBPJ alone in IPCs (cluster 3) (Fig. 3a–c). To confirm that these results were not due to expression of NICD, rather than full length NOTCH, we performed TaDa with full length NOTCH (NOTCH-FL) and compared these results with NICD. We found the binding patterns of NOTCH-FL and NICD in RGCs and IPCs to be remarkably consistent across the 8 clusters (Supplementary Fig. 7).

To assess whether chromatin state might influence NOTCH/RBPJ binding, we assayed chromatin accessibility using an untethered Dam methylase (CaTaDa^15,30^; Fig. 3d) and compared our results with previously published ATAC-seq results^32^. Clusters where NOTCH and RBPJ co-localized (clusters 2, 4, 6, 7), were more accessible than those where only one of the two factors were bound (clusters 1, 3, 5, 8) (Fig. 3d; Supplementary Fig. 8a). Comparative motif analysis with i-CisTarget^47^ revealed overall high similarity between most peak clusters (Supplementary Fig. 8b) and a strong overlap with embryonic brain-specific regulatory regions (ENCODE^31^) (Supplementary Fig. 8c). In contrast, the binding sites with the lowest average accessibility (clusters 3 and 8) (Fig. 3d; Supplementary Fig. 8a) were associated with a different set of motifs and genomic features than the other clusters (Supplementary Fig. 8b,c). These peaks are characterized by RBPJ binding without Notch (Fig. 3a–c) and are reminiscent of previously identified constitutive RBPJ binding sites^20,48^. This may point to a role for RBPJ in maintaining transcriptional repression or, conversely, as part of a pioneering complex to unmask enhancers during differentiation. Interestingly, RBPJ binding motifs could not be detected under the IPC^RBPJ^-specific peaks (cluster 3) (Supplementary Fig. 8d) suggesting that other factors, possibly homeobox transcription factors (Fig. 2e; Supplementary Fig. 8d), may recruit RBPJ to regulate target gene expression upon differentiation. Together, these data suggest^2,45,49^ that, in parallel to NOTCH/RBPJ-binding, differences in chromatin accessibility and co-factor occupancy may influence NOTCH/RBPJ-target gene expression (Fig. 3e).

### Notch/RBPJ are able to bind at NSC-genes when expressed ectopically in intermediate progenitors

**Fig. 4:**
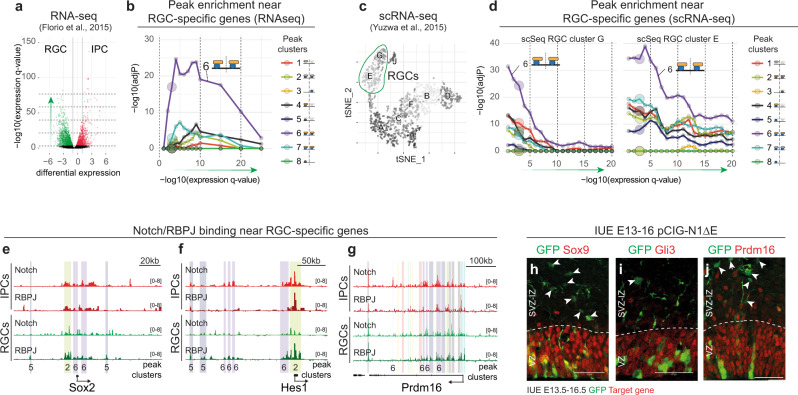
Ectopic NICD can bind to RGC-specific genes during differentiation. **a**–**d** Bulk RNA-seq^34^ **a** and single-cell RNA-seq^35^ **c** of RGCs and IPCs. Enrichment of NOTCH/RBPJ peak clusters **b**, **d** near genes differentially expressed in RGCs as compared to IPCs. **e**, **f**, **g** NOTCH and RBPJ binding profiles in RGCs and IPCs near RGC-specific genes. Peak loci and corresponding peak cluster (coloured shading corresponds to clusters in Fig. 3) shown. **h**–**j** IUE of pCIG-N1ΔECD stained for GFP (green) and the indicated RGC-specific NOTCH/RBPJ target gene (*Sox9*, *Gli3*, *Prdm16*; red; *n* = 1). Arrowheads indicate differentiating electroporated cells in the SVZ/IZ. Scale bars 50 µm.

**Fig. 5:**
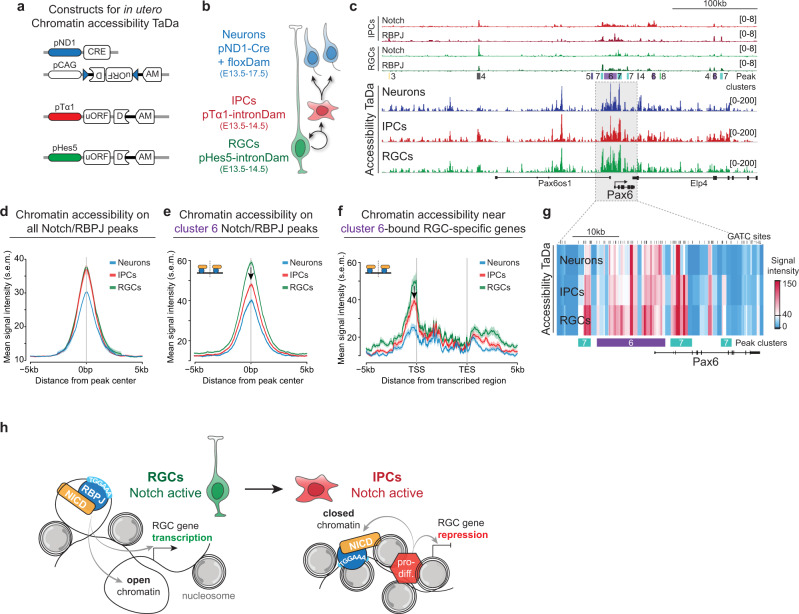
Chromatin accessibility correlates with NOTCH target gene expression. **a**, **b** Constructs for *in utero* chromatin accessibility TaDa and cell type specific expression pattern. **c** Chromatin accessibility profiles at the *Pax6* locus (bottom), and the corresponding NOTCH/RBPJ binding profiles (top). **d**–**f** Accessibility (average signal ± s.e.m., shaded area) at all NOTCH/RBPJ peak regions **d** on NOTCH/RBPJ cluster 6 peaks **e** and on cluster 6-bound RGC-specific genes **f** in the indicated cell types. **g** Heatmap of accessibility in the region highlighted in (**c**) colour scale indicates TaDa binding intensity. GATC sites and NOTCH/RBPJ peaks are indicated. **h** Model of NOTCH target gene repression during differentiation after ectopic expression of NICD-Dam.

When NOTCH/RBPJ were bound together in RGCs, they could also be detected after ectopic expression in IPCs, where they mostly co-localized (5520/7560, 73%; clusters 2, 6, 7) (Fig. 3a–c). Genes near these peaks showed the strongest enrichment for GO terms related to neurogenesis and Notch signalling (Supplementary Fig. 9a, b). This could suggest that NOTCH/RBPJ may be able to bind at NOTCH-regulated NSC-genes during differentiation. To determine on a genome-wide basis how NOTCH/RBPJ binding dynamics correlate with transcriptional changes during neurogenesis, we intersected the eight NOTCH/RBPJ peak clusters with published RNA-seq data from sorted RGCs and IPCs at E13.5^34^ (Fig. 4a, b; Supplementary Fig. 10a–e), and with two independent single-cell RNA-seq datasets^35,50^ at E13.5 and E14.5 (Fig. 4c, d; Supplementary Fig. 10f–q). Peaks with constitutive NOTCH/RBPJ binding potential (cluster 6) were highly enriched near genes specifically expressed in RGCs (Fig. 4b, d; Supplementary Fig. 10c, k, q). The RGC-specific genes *Sox2*, *Pax6* and *Hes1* (see Fig. 1k–m) were all associated with peaks assigned to cluster 6 (Figs. 4e, f and 5c). In contrast, genes that were more highly expressed in IPCs (Supplementary Fig. 10d, k, q) or neurons (Supplementary Fig. 10e, k, q) were mostly near IPC^RBPJ^-specific binding sites (cluster 3).

The strong enrichment of cluster 6 peaks, NICD and RBPJ binding in RGCs and IPCs near genes that are downregulated upon differentiation, was surprising given that downregulation of NOTCH-target genes is thought to result from the loss of NICD binding^51^. 131 genes that were shown to be expressed specifically in RGCs^37^ (FDR < 10^−5^) were associated with cluster 6 peaks (Supplementary Data 4). The regions associated with cluster 6 peaks could also be bound in IPCs by full-length NOTCH, as assayed by TaDa (Supplementary Fig. 7b–e). It appears that NOTCH, when expressed ectopically in IPCs, is able to bind to RGC-specific genes that are not normally expressed in this cell type, such as *Hes1* and *Pax6* (Fig. 1k–m). The RGC-specific genes encode many well-known transcriptional regulators of RGC fate and stem cell maintenance, such as *Prdm16*^52^, *Sox9*^53^ and *Gli3*^54^. Of these, almost half (48.7%) showed significantly increased expression upon ectopic NICD expression in the embryonic cortex, as documented previously by RNAseq^37^ (p < 0.05 and fold-change >1.5); Supplementary Data 4). This suggests that, at least within NSCs, the genes can be activated by ectopic NICD. In our hands, expression of NICD-Dam in IPCs did not block the downregulation of RGC genes such as *Sox9*, *Gli3* and *Prdm16* (Fig. 4h–j), suggesting that other factors may help to repress RGC genes in IPCs, even in the presence of activated NOTCH, and thereby enable neuronal differentiation.

### NSC genes bound by NOTCH/RBPJ become inaccessible upon differentiation

To assess whether changes in chromatin accessibility during differentiation correlate with differential expression of NOTCH/RBPJ target genes, we assayed cell-type specific chromatin accessibility in vivo in RGCs, IPCs and postmitotic neurons by *in utero* TaDa with untethered Dam (Fig. 5a, b)^15,30,33^. This generated cell-type specific accessibility profiles comparable to whole-brain ATAC-seq^32^ (Supplementary Fig. 11a, b). Surprisingly, the profiles showed strong similarity between different cell types, particularly between RGCs and IPCs (Fig. 5c). Most NOTCH/RBPJ peak clusters also did not show dynamic changes in chromatin accessibility between RGCs and IPCs (Fig. 5d; Supplementary Fig. 11d, f, g). On cluster 4 peaks, for example, which have decreased binding of NOTCH/RBPJ upon differentiation, average Dam-accessibility was indistinguishable between RGCs and IPCs, and only decreased upon differentiation into postmitotic neurons (Supplementary Fig. 11f). This demonstrates that chromatin accessibility and cell-type specific binding dynamics of NOTCH/RBPJ appear to be regulated independently.

In contrast, near RGC-specific genes, the average accessibility on the Notch/RBPJ co-binding sites (cluster 6) decreased between RGCs and IPCs (Fig. 5e, f; Supplementary Fig. 11f–h), as did accessibility across the upstream regulatory regions of NOTCH/RBPJ-bound genes (Fig. 5f; Supplementary Fig. 11e). This suggests that chromatin accessibility may help to restrict Notch activity during neurogenesis, for example, at loci where NOTCH/RBPJ might still be bound.

## Discussion

Intercellular signalling is mediated by signalling pathways that are used iteratively throughout development and disease. How the same signal is interpreted differently depending on cellular context remains largely unanswered. This has been due, in part, to the need for sensitive tools for cell type specific genome-wide chromatin profiling in vivo. Targeted DamID enables genome-wide profiling of DNA- or chromatin-binding without cell isolation, fixation or affinity purification^13,15–18^. Using vectors that overcome background plasmid methylation that can interfere with transient transfection experiments, we were able to achieve genome-wide profiling in limiting cell numbers in vivo, without disruption of the native tissue environment. We profiled NOTCH and RBPJ binding in RGCs and IPCs in the developing mouse cerebral cortex after IUE. We found that NOTCH/RBPJ binding patterns in specific cell types correlate with chromatin accessibility although we also found many putative binding sites of NOTCH or RBPJ in relatively inaccessible chromatin regions. Enrichment of homeobox transcription factor binding motifs at NOTCH/RBPJ sites suggests a role for transcription factors like LHX2^44^ in establishing a permissive chromatin environment to enable a tissue specific response to Notch, in a manner similar to that shown for Runx transcription factors in *Drosophila*^49^ and T-cell leukemia^48^.

To assess the transcriptional activity of the putatively bound loci, we screened publicly available cell-type specific transcriptional data sets^34,35,50^ and found that ectopically expressed NOTCH and RBPJ could bind, perhaps only transiently, to RGC-specific genes in IPCs. Therefore, additional mechanisms may ensure that RGC-specific NOTCH target genes are inactivated to enable neurogenesis if NOTCH were to remain present during or after the transition of RGC to IPC. One such mechanism could be a reduction in chromatin accessibility at the regulatory regions of Notch target genes which, when paired with a decrease in NOTCH/RBPJ expression, could facilitate efficient repression of RGC-specific genes. Nevertheless, given that NOTCH/RBPJ can bind their target genes in IPCs, we hypothesize that the binding events occurring in RGCs could be maintained in IPCs, making chromatin accessibility changes essential for blocking the activation of RGC-specific NOTCH target genes.

Our data represent a population level view of NOTCH activity and it remains to be determined whether there is heterogeneity of NOTCH/RBPJ binding within cell types at the single cell level. With recent advances in single-cell technology, future experiments may be able to better characterize variability in NOTCH activity within cell populations in the developing cortex. The factors that determine the competence of cells to respond to Notch pathway activation have broad relevance, not only to the multitude of developmental processes in which Notch is involved, but also to pathologies such as cancers. Chromatin accessibility and the tumour cell of origin within the NSC lineage^55^, may influence the progression of specific brain tumour subtypes^11,12^.

## Methods

### Constructs

#### Intron-dam constructs

For intron1Dam, the sequence of intron 3 of mouse IghE^56^ was inserted between helix 3 and 4 of the DNA-binding domain of the Dam methylase^57^ in pPB-PGK-mcherry-Dam^17^. mCherry-intron1Dam-SV40polyA was cloned into pCAG-IRES-GFP (pCIG, gift from P. Vanderhaeghen) to give pCAG-mcherry-intron 1Dam. For intron2Dam a modified version of the Promega chimeric intron sequence^58–60^ was subcloned into pCAG-mcherry- intron1Dam, replacing intron 1 and modifying the exon junctions. intron2Dam was more efficient, as assessed by RT-PCR, and was used for most experiments.

#### FloxDam construct

Lox71 and Lox61 sites^61^ were inserted into the NheI site upstream of mCherry and the XmaI site inside intron2, respectively. The two Lox-sites and intervening sequence were then inverted to give pCAG-flox2Dam.

#### Promoter-fragments

A 764 bp fragment of the mouse Hes5-promoter and 5’UTR^38^, and a 1097 bp fragment of the mouse Tuba1a promoter, Tα1^4,39,40^, were amplified from genomic DNA and cloned into SpeI-HindIII cut pCAG-mcherry-intron2Dam. pHes5-d2eGFP was a gift from R. Kageyama. IRES-GFP was removed from pND1-IRES-GFP (gift from F. Polleux) and replaced with Cre to generate pNeurod1-Cre. pTα1-mcherry-NLS-DR was cloned by Gibson assembly from Addgene plasmid 84603^62^. pCAG-Venus was generated by removing the U6-shRNA cassette from pSCV2 (gift from F. Polleux).

#### Ectopic Notch expression

For the IUE experiments with constitutively active Notch the plasmid pCIG-NΔECD was used, which consists of pCAG-NΔECD-IRES-GFP and was a gift from G. Del Sal^63^. As a control, an empty pCAG-IRES-GFP plasmid was used (pCIG, gift from P. Vanderhaeghen).

#### Dam-fusion proteins

The mouse Notch1 intracellular domain with the transmembrane domain (NΔECD) or without the transmembrane domain (NICD) was amplified from pCIG-NΔECD (pCS-NΔECD, gift from G. Del Sal^63^). Mouse full-length NOTCH was from Addgene 41728^64^. hRBPJ1 was amplified from cDNA of human ESC-derived NSCs. mCherry(2), mammalian codon-optimized version of mCherry, was from Addgene 84603^62^. Plasmids for IUE were prepared from Dam-negative bacteria with Endofree Plasmid Maxi kit (Qiagen 12362). Molecular weight ladder is Hyperladder 1 kb (Bioline BIO-33026).

### *in utero* electroporation

All mouse husbandry and experiments were carried out in a Home Office-designated facility, according to the UK Home Office guidelines upon approval by the local ethics committee (project licence PPL70/8727). Experiments were done in wild-type MF1 mice. Timed natural matings were used, where noon of the day of plug-identification was E0.5. IUE was performed as previously described^65,66^ at E13.5 with 50 ms, 40 V unipolar pulses (BTX ECM830) using CUY650P5 electrodes (Sonidel). DamID plasmids were injected at 1 µg/µl together with pCAG-Venus at 0.25 µg/µl. All other plasmids were injected at 1 µg/µl. Embryos were harvested after 24 hours (E14.5) for TaDa with pHes5 and pTα1, after 72 hours (E16.5) for TaDa with pND1-Cre and after 96 hours (E17.5) for TaDa with pCAG-intronDam. For RGC^Notch^ data, we combined samples from pHes5-mCherry-NICD-intron2Dam, pHes5-mCherry(2)- intron2Dam and pHes5-mCherry-NdE-intron2Dam. IUE of pHes5-NΔECD-intron2Dam did not prevent differentiation, consistent with the very low levels of translation of the Dam-fusion protein^13,17,28^.

### Immunostaining and imaging

For immunostaining, embryos and tissue were processed as previously described^24^ and staining was performed on 100 µm thick vibratome sections in PBS with 0.3% Triton (PBST) and 3% BSA (Sigma A3608). Antisera were as follows: chicken anti-GFP 1/1000 (Abcam ab13970), rabbit anti-RFP 1/500 (Abcam ab62341), goat anti-Sox2 1/500 (R&D AF2018), rabbit anti-Sox9 (Millipore AB5535), rabbit anti-Prdm16 1/200 (gift from P. Seale^67^), rabbit anti-Pax6 1/500 (Covance PRB-278P), rabbit anti-Hes1 1/100 (Cell Signalling D6P2U), goat anti-Gli3 1/500 (R&D AF3690). DNA was stained with DAPI. Fluorescent images were acquired using a Leica SP8 confocal microscope and analysed using ImageJ.

### DamID-seq

For DamID after IUE, embryos were cooled on ice, and the electroporated cortex was identified with a fluorescent binocular microscope. Meninges were removed and the electroporated region microdissected. Tissue was then processed for DamID as described previously^15^. DamID fragments were prepared for Illumina sequencing according to a modified TruSeq protocol^15^. All sequencing was performed as single end 50 bp reads generated by the Gurdon Institute NGS Core using an Illumina HiSeq 1500.

### DamID-seq data processing

Quality check of *.fastq-files was performed with FastQC (v0.11.5), reads were trimmed with TrimGalore (v0.4.5) and if necessary deduplicated with the Clumpify tool of the BBmap suite (v38.12; dedupe subs=0). Data processing was automated and parallelized by using workflow scripts interacting with the slurm workload manager (v15.08.13). Preprocessed *.fastq-files were mapped to the mm10 (GRCm38.p6) genome assembly. Reads were binned into fragments delineated by 5’-GATC-3’ motifs (GATC-bins). Individual replicates for the Dam-fusion constructs (3× pHes5-iDam-RBPJ; 4× pHes5-Notch-iDam; 3× pTa1-iDam-RBPJ; 4× pTa1-Notch-iDam; 4× pHes5-NotchFL-iDam; 2× pTa1-NotchFL-iDam) were normalized against separate Dam-only replicates (7× pCAG-iDam; 5x pHes5-iDam; 4× pTa1-iDam, 3× pCAG-floxDam) with a modified version of the damidseq pipeline^14^ (RPM normalization, 300 bp bins) and all resulting binding profiles for one Dam-fusion construct were quantile normalized to each other^14^. The resulting logarithmic profiles in bedgraph format were averaged for all GATC-bins across the genome and subsequently backtransformed (“unlog”). In parallel, non-normalized Dam-only scores for all GATC-bins were provided separately by the modified damidseq_pipeline as a means to assay chromatin accessibility^30^. Files were converted to the bigwig file format with bedGraphToBigWig (v4) for visualization with the Integrative Genomics Viewer IGV (v2.4.19).

### Peak calling, peak-gene association and overlap with genomic features

Macs2 (v2.1.2)^68^ was used to call broad peaks for every dam-fusion/dam-only pair on the set of *.bam-files generated by the damidseq_pipeline, using Dam-only as control. Peaks were filtered stringently for FDR < 10^−5^ and were only considered if present in all pairwise comparisons for a particular experimental condition (i.e. all replicates from a Dam-fusion construct in one cell type). Relevant Ensembl gene and genomic feature annotations for mm10 were acquired via biomaRt (v2.38.0). Peaks were annotated to genes with bedtools (v2.26.0) by identifying the closest TSS of a protein coding gene along the linear genome. Upstream sequences were defined as regions covering 5 kb upstream of TSS of all annotated transcripts from protein coding genes. Intergenic regions are the areas outside protein coding genes (including 5 kb upstream sequence, 5’UTR, 3’UTRs, introns and exons). To avoid artificial inflation of associations, overlapping regions were collapsed in a feature-wise manner via the bedr R package (v1.0.7) and bedtools (v2.26.0). Given the inherent ambiguity of genomic features, particular genomic areas can be part of two features. Associating peaks with the defined genomic features was performed using gat (v1.3.2) with entire chromosomes specified as workspace and 1 mio computed samples. The relative overlap of peaks with genomic features normalized for the combined length of all included peaks (percent_overlap_size_track) was visualized. Peaks from ATAC-seq in E13.5 forebrain (ENCFF798QON.bam^31^) were called with Macs2 (v2.1.2) using the same broad peaks settings as above but without control. Raw data from RBPJ ChIP-seq in mouse cortex^37^ was not publicly available, but peaks were extracted from the Supplementary Information, coordinates converted to mm10 and genes called as described above.

### Peak clustering

Overlapping peaks derived from different Dam-fusion constructs were merged into consensus peaks via bedtools (v2.26.0). Averaged binding intensities were normalized for length of the consensus peak region, converted to z-scores and subjected to unsupervised clustering to identify stable binding patterns. The optimal cluster number was determined with the help of the R packages factoextra (v1.0.5), clValid (v0.6-6) and mclust (v5.4.5). k-means as the clustering approach was chosen and its parameters optimised by calculating silhouettes with the R cluster package (v2.0.7-1). Dimensionality reduction for visualizing the distribution of clustered peaks was conducted with the Rtsne package (Rtsne v0.15, perplexity = 100, theta = 0).

### Motif detection

Comparative motif analysis was performed with i-cisTarget^47^. Enriched features were detected by uploading coordinates for all peaks of individual Notch/RBPJ k-means clusters to the i-cisTarget online platform (Gene annotation: RefSeq r70, Database: v5.0) after conversion to mm9 with the liftOver function of rtracklayer (v1.42.2). Normalized enrichment scores across all features and all k-means clusters were converted to z-scores, subjected to hierarchical clustering by calculating a matrix of euclidean distances with the hclust (method = complete) and dist commands of the R stats package (v3.6.1) and visualized by using the ggdendro package (v0.1.20). The branch depth was reduced to 4 for simplicity with the cut function of the stats package and the R dendextend package (v1.10.0). De novo motif analysis on binding sites was performed with Homer^69^ (findMotifsGenome.pl) and motifs consisting of 5’-GATC-3’ were manually removed from the results. Known motif enrichment was analysed with AME of the MEME suite with the following scoring method: Average odds score; Fisher’s exact test, E-value ≤ 1808; Motif databases: Jaspar Core Vertebrates non-redundant, UniProbe Mouse, Jolma2013 Human and Mouse. Sequences corresponding to all peaks were first retrieved from the Biostrings genome object for the UCSC mm10 genome (BSgenome.Mmusculus.UCSC.mm10, v1.4.0) with the getSeq command of the Biostrings R package (v2.50.2).

### Comparative GO term analysis

Gene Ontology term enrichment of genes associated with peaks was done using the broadenrich command of chipenrich (v2.10.0) for all available mm10 genesets in chipenrich.data (v2.10.0) (locusdef = nearest_tss). Gene identifiers were converted with AnnotationDbi (v1.48.0) based on the org.Mm.eg.db (v3.10.0) database. Enrichment of GO terms annotated to biological processes (GO_BP) was visualized by plotting -log10-transformed p-values, if the calculated p-value was <0.01 in at least one of the compared conditions or clusters. GO terms belonging to manually curated lists of keywords associated with indicated signalling pathways or biological processes were highlighted in the same colors in bar- and violin/dot-plots.

### Genome wide correlation

Mapped reads (see DamID-seq data processing) were extended to 150 bp with bamCoverage from the deepTools suite (v3.1.3). The resulting *.bedgraph files were read into R, reads binned into 500 bp bins or into the regions covered by all NOTCH/RBPJ peaks and Pearson Correlation Coefficients were calculated with the cor command of the R stats package (v3.6.1). Coefficients for all pairwise comparisons were plotted as tiled heatmap.

### Bulk RNAseq data processing

Bulk RNAseq datasets for mouse cortex cell types^34^ were acquired from GEO (GSE65000) and trimmed with TrimGalore (v0.4.5). *.fastq.gz files were pseudoaligned with kallisto (v0.45.0;-b 100–single -l 200 -s 30) and statistical analysis was performed with sleuth (v0.30.0) by specifying full models for pairwise combinations of cell types. Reads were aggregated per gene rather than transcript while preparing the sleuth object with the sleuth_prep command (i.e., gene_mode=TRUE). Significance of differential expression was determined via q-values derived from wald tests (sleuth_wt) based on the full model. Raw data from bulk RNAseq upon NICD overexpression^37^ was not publicly available, but genes with a p-value for differential expression <0.05 were extracted from the Supplementary Information.

### Single cell RNAseq data processing

Single cell RNAseq datasets derived from mouse cortices at E13.5^35^ and E14.5^50^ were sourced from GEO as raw count matrices with the accession number (GSE107122 preselected for cortex-only cell types; GSE123335 combined matrix). GSE107122 (E13.5) was read into R as a Seurat Object via the corresponding R package Seurat (v2.3.4; min.cells = 3, min.genes = 200). Cells were filtered for maximal 4800 genes per cell and an upper threshold of 8% reads allocated to mitochondrial genes, the data log-normalized (scale.factor = 100000), scaled and centred dependent on nUMI and percent of reads aggregated on mitochondrial genes. Dimensionality reduction via PCA preceded clustering of cells using 5 dimensions as evaluated by JackStraw analysis. The Wilcoxon rank sum test was used to identify significantly differentially expressed genes for every cluster compared to all other clusters or for sets of clusters annotated to their respective cell types (IPCs, RGCs, neurons) via the FindAllMarkers command (logfc.threshold = 0, min.pct = 0, only.pos = FALSE, return.thresh = 1). GSE123335 (E14.5) was read into R as a dgCMatrix via the methods package (v3.6.1) and processed by creating a Seurat Object with Seurat (v3.0.2; min.cells = 3, min.genes = 200). To properly integrate all 6 replicates combined in the matrix, they were first separated via SplitObject, subsequently normalized via NormalizeData and further prepared for integration via Canonical Correlation Analysis with FindVariableFeatures and FindIntegrationAnchors^70,71^. To avoid detection of differentially expressed genes, nfeatures was set to 18361 corresponding to the number of genes detected across all 6 scRNAseq replicates. Dimensionality reduction was performed with the RunPCA and RunTSNE commands based on 90 dimensions. Differential expression analysis was performed cluster-wise as indicated for GSE107122 data with the FindMarkers command.

### Statistical modelling of peak-expression correlation

Associations between sets of peaks and significantly differentially expressed genes from bulk RNAseq or scRNAseq data were modelled as binomial distributed, since multiple peaks can be linked to the same gene. Based on the assumption that individual peaks have the highest likelihood to associate with the gene whose TSS is closest, associations of significantly and non-significantly differentially expressed genes with peaks as well as the total number of significantly and non-significantly differentially expressed genes were summed at a particular significance threshold (q-value, adjusted p-value). To test for enrichment of peak-to-gene associations, the binom.test command of the R stats package (v3.6.1) was used (alternative = less, conf.level = 0.95). This test was repeated across multiple gene expression significance thresholds and peak sets (cell-types, constructs, clusters). The resulting p-values were adjusted due to multiple testing with the Bonferroni correction as implemented in the p.adjust command of the R stats package (v3.6.1, method = bonferroni). Adjusted p-values and the number of peaks associated with expressed genes (peaksAtGenes) were visualized as a function of the significance threshold of differentially expressed genes (-log10 transformed).

The overlap between bulk RNAseq data derived from mouse cortices with NICD-overexpression^37^ and genes associated with RPBJ-peaks from either DamID-seq or RBPJ ChIP-seq (upon NICD overexpression)^37^ was modelled as hypergeometrically distributed. To assess the background overlap level between peaks and differentially expressed genes, the same number of randomly chosen genes as associated with the corresponding peaks were sampled from the mm10 genome (GRCm38.p6). Sampling was repeated 1000 times for both sets of peaks in the same manner and p-values were calculated with the phyper function of the R stats package (v3.6.1, lower.tail = FALSE).

### Data visualization

Genome browser views were generated using the Integrative Genomics Viewer IGV (v2.4.19)^72^ with the midline for TaDa ratio tracks set at 1 and for ATAC-seq set at 0. Peak or gene coordinates were saved in *.bed format and supplied as features to the getPlotSetArray command of Seqplots (v1.12.1)^73^. Quantile normalized, averaged and backtransformed TaDa profiles or quantile normalized chromatin accessibility profiles were provided in bigwig format. plotAverage and plotHeatmap were used to visualize and average the binding intensities across all supplied coordinates. Signal from the mitochondrial genome, the IghE-intron, the Hes5-, Tuba1a- and NeuroD1-promoters were removed for average plots of chromatin accessibility TaDa. Statistical analysis of chromatin accessibility in RGCs and IPCs was done with a Kruskal Wallis test and a post-hoc pairwise Wilcoxon rank sum test. Plasmid maps were generated using SnapGene. Figures were assembled in Adobe Illustrator.

### Reporting summary

Further information on research design is available in the Nature Research Reporting Summary linked to this article.

## Supplementary information

Supplementary Information

Description of additional Supplementary File

Supplementary Data 1

Supplementary Data 2

Supplementary Data 3

Supplementary Data 4

Reporting Summary

## Data Availability

The data that support this study are available from the corresponding author upon reasonable request. Raw and processed sequencing data of all datasets in this study have been deposited on NCBI GEO under the accession code GSE152207. ATAC-seq data from E13.5 mouse brain was obtained from the ENCODE portal (ENCFF450ZSN.bigWig for p-values, ENCFF798QON.bam for alignments)^31^. Single cell and bulk RNAseq datasets were obtained from GEO (GSE107122^35^, GSE123335^50^ and GSE65000^34^). Raw data from RBPJ ChIP-seq and RNA-seq upon NICD overexpression in mouse cortex^37^ are not available anymore, but peak information was obtained from their Supplementary Table 5 (https://academic.oup.com/stmcls/article/30/4/741/6415703) and differential gene expression from their Supplementary Table 1 (https://academic.oup.com/stmcls/article/30/4/741/6415703). Source data are provided with this paper.

## Source data

Source Data

## Code availability

Scripts covering the outlined analyses are available on request.
