# Supplementary material for: Reduced chromatin accessibility correlates with resistance to Notch activation
Source: Nat Commun. 2022 Apr 25;13:2210. doi: 10.1038/s41467-022-29834-z (PMC9039071; doi:10.1038/s41467-022-29834-z)
Supplement: Supplementary file 1 — Supplementary Information [file 41467_2022_29834_MOESM1_ESM.pdf]

Supplementary Figures

Reduced chromatin accessibility correlates with resistance to Notch activation

Jelle van den Ameele, Robert Krautz, Seth W. Cheetham, Alex P.A. Donovan, Oriol Llorà-Batlle, Rebecca Yakob and Andrea H. Brand

Supplementary figures

- Supplementary Fig. 1. *In vivo* Targeted DamID (TaDa) constructs.
- Supplementary Fig. 2. *In utero* Targeted DamID.
- Supplementary Fig. 3. Genome-wide Pearson correlation matrices.
- Supplementary Fig. 4. Cre-inducible construct (floxDam).
- Supplementary Fig. 5. Cell-type specific *in utero* TaDa of Notch and RBPJ.
- Supplementary Fig. 6. k-means clustering for Notch/RBPJ peak regions.
- Supplementary Fig. 7. TaDa in RGCs and IPCs with full-length Notch.
- Supplementary Fig. 8. Properties of Notch/RBPJ peak clusters.
- Supplementary Fig. 9. Gene ontology analysis of NOTCH/RBPJ-bound clusters.
- Supplementary Fig. 10. Genome-wide comparison of cell-type specific expression data and Notch/RBPJ binding patterns.
- Supplementary Fig. 11. Cell-type specific *in utero* chromatin accessibility TaDa.

Supplementary Data files

- Supplementary Data 1. Loci bound by NOTCH/RBPJ in RGCs or IPCs and their associated genes. Related to Fig. 2.
- Supplementary Data 2. Overlap of genes bound by RBPJ ChIP-seq, RGC-specific RBPJ TaDa and RNAseq upon NICD overexpression. Related to Supplementary Fig. 5g.
- Supplementary Data 3. Peaks associated with each Notch/RBPJ peak cluster and the associated gene for each peak. Related to Fig. 3.
- Supplementary Data 4. RGC-specific genes determined by bulk RNA-seq that are bound by NOTCH/RBPJ in peak cluster 6. Related to Fig. 4b.

Source Data file

- Source data Fig1j-SupplFig2d.xlsx

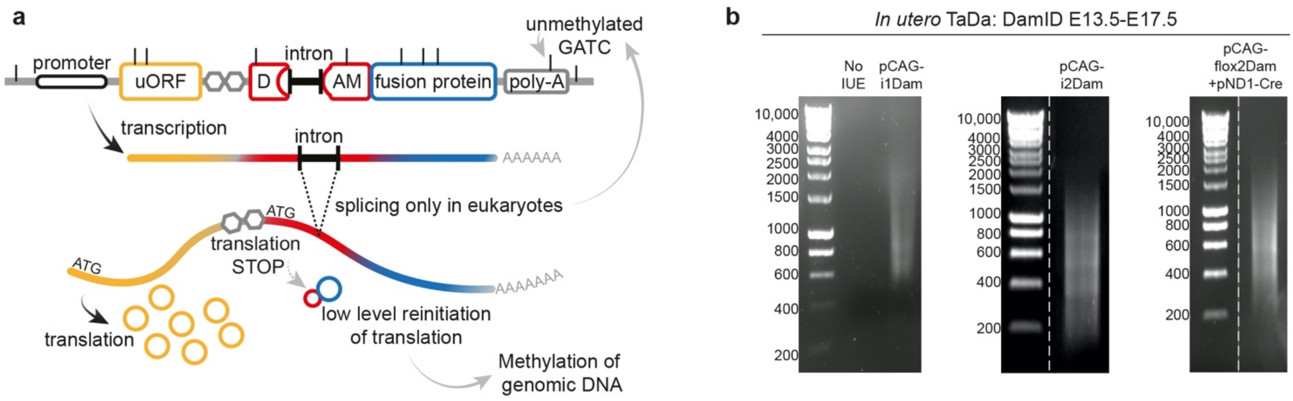

Supplementary Fig. 1. *In vivo* Targeted DamID (TaDa) constructs. a. Schematic of TaDa construct, where an upstream open reading frame (uORF) followed by two stop codons and a frame shift (5’-TAATAAC-3’) reduces translation of the downstream open reading frame encoding a Dam fusion protein [13]. Introduction of an intron in the Dam coding sequence prevents expression in bacteria. **b**. TaDa of E17.5 cortex electroporated at E13.5 with the indicated constructs: pCAG-Venus only (lane 1, n=2) or together with pCAG-i1Dam (lane 2, n=3), pCAG-i2Dam (lane 3, n=4) and pCAG-flox2Dam with pNeuroD1-Cre (lane 4, n=3). Ladder is Hyperladder 1 (Bioline).

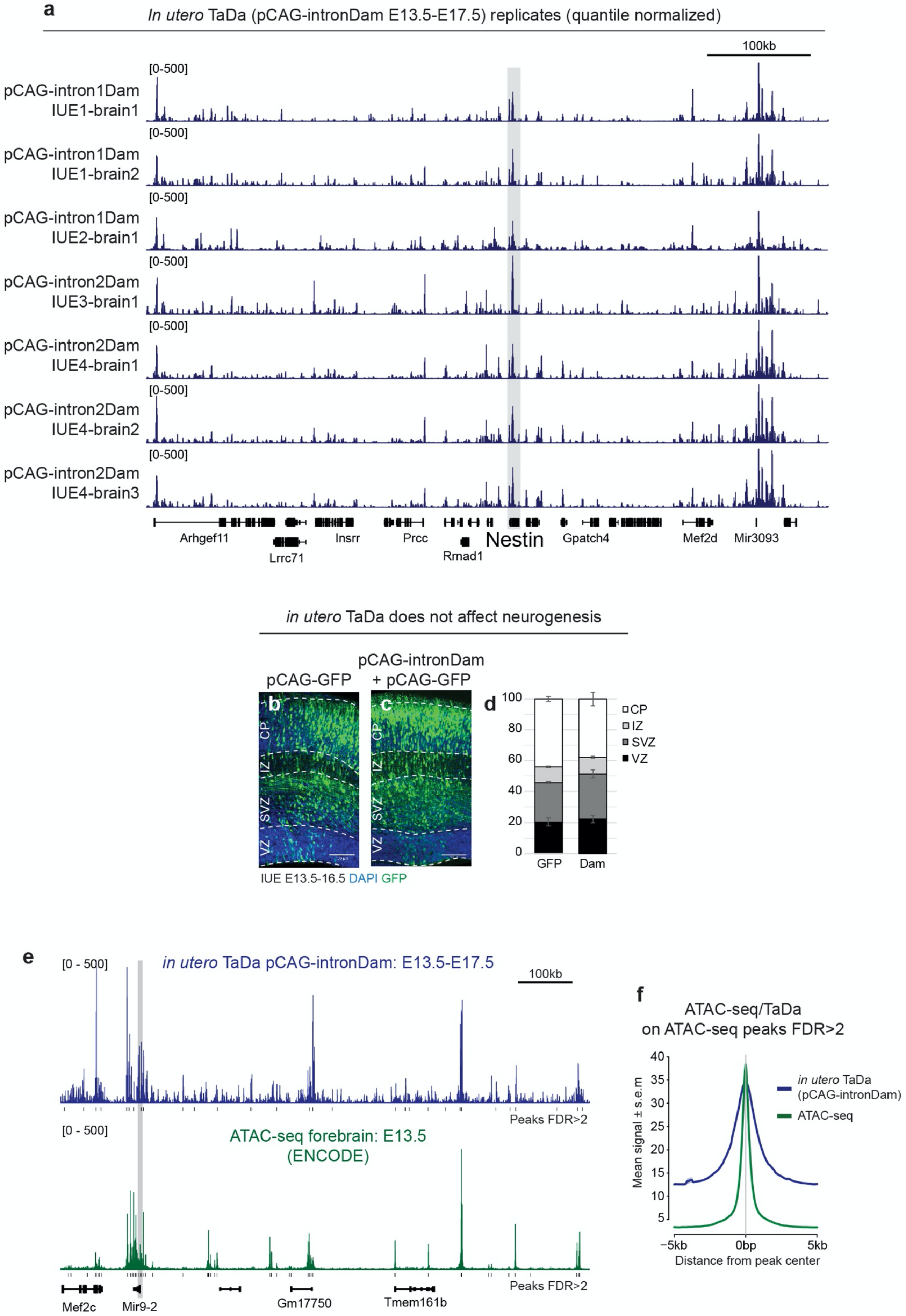

Supplementary Fig. 2. *In utero* Targeted DamID. a. Chromatin accessibility profiles in the genomic region surrounding the *Nestin* locus (grey shading) of individual pCAG-mCherry-i1/2Dam

replicates from 7 embryos across 4 litters harvested 96 hours (E17.5) after IUE at E13.5. **b-d**. *In utero* electroporation of pCAG-Venus either alone (**b**) or with pCAG-mCherry-i2Dam (**c**) at E13.5. Coronal sections stained for DAPI (nuclei, blue) and GFP (green) 3 days after IUE (E16.5). Dashed lines mark the VZ, SVZ, IZ and CP. Scale bars are 100µm. Histograms showing the percentage of GFP^+ cells in each indicated region (**d**). Data are presented as mean ± s.e.m. (n = 3 for pCAG-Venus and 2 for pCAG-i2Dam embryos). Source data are provided as a Source Data file. **e,f**. Forebrain ATAC-seq profiles at E13.5 from ENCODE and *in utero* TaDa with pCAG-mCherry-iDam at the *mir9-2* locus (grey shading) (**e**) and mean signal ± s.e.m. (shaded area) plotted over all ATAC-seq peaks at $FDR<10^{-2}$ (**f**).

a

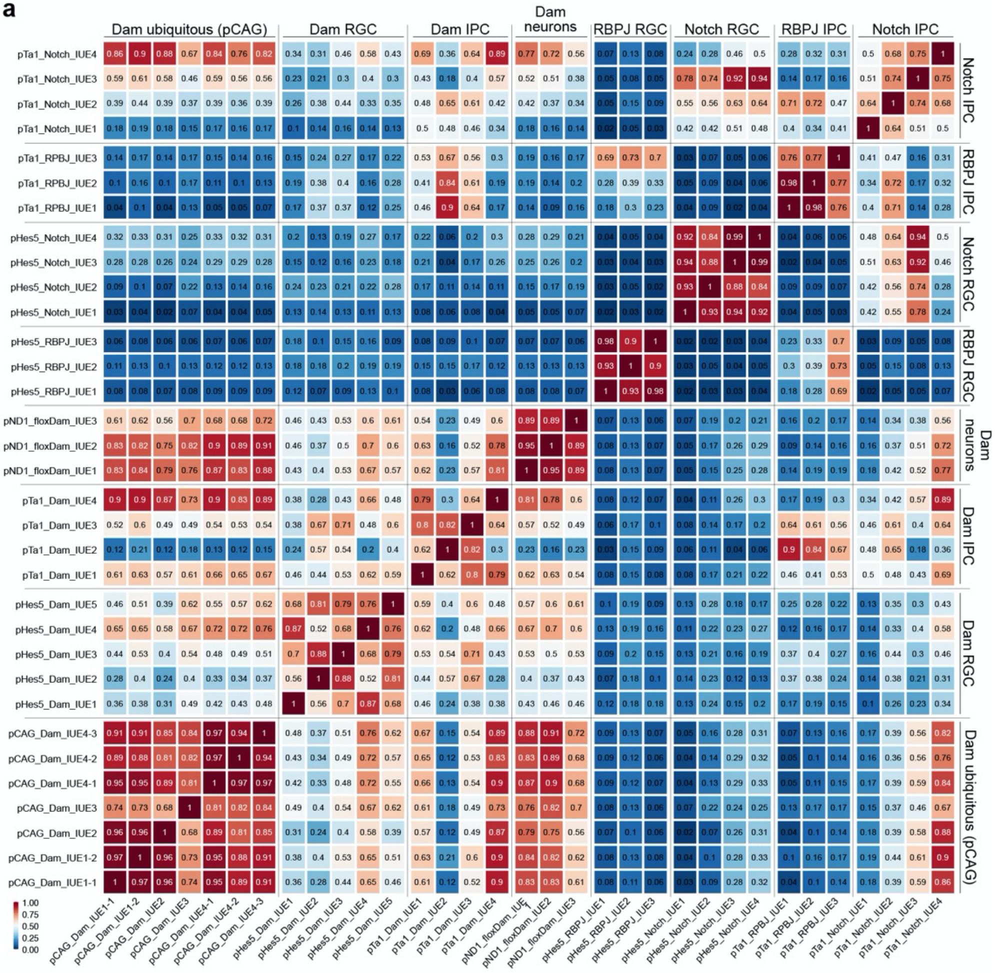
Dam ubiquitous (pCAG)
Dam RGC
Dam IPC
Dam neurons
RBPJ RGC
Notch RGC
RBPJ IPC
Notch IPC
pTa1_Notch_IUE4
pTa1_Notch_IUE3
pTa1_Notch_IUE2
pTa1_Notch_IUE1
pTa1_RPBJ_IUE3
pTa1_RPBJ_IUE2
pTa1_RPBJ_IUE1
pHes5_Notch_IUE4
pHes5_Notch_IUE3
pHes5_Notch_IUE2
pHes5_Notch_IUE1
pHes5_RBPJ_IUE3
pHes5_RBPJ_IUE2
pHes5_RBPJ_IUE1
pND1_floxDam_IUE3
pND1_floxDam_IUE2
pND1_floxDam_IUE1
pTa1_Dam_IUE4
pTa1_Dam_IUE3
pTa1_Dam_IUE2
pTa1_Dam_IUE1
pHes5_Dam_IUE5
pHes5_Dam_IUE4
pHes5_Dam_IUE3
pHes5_Dam_IUE2
pHes5_Dam_IUE1
pCAG_Dam_IUE4-3
pCAG_Dam_IUE4-2
pCAG_Dam_IUE4-1
pCAG_Dam_IUE3
pCAG_Dam_IUE2
pCAG_Dam_IUE1-2
pCAG_Dam_IUE1-1
Notch IPC
RBPJ IPC
Notch RGC
RBPJ RGC
Dam neurons
Dam IPC
Dam RGC
Dam ubiquitous (pCAG)
1.00
0.75
0.50
0.25
0.00

b

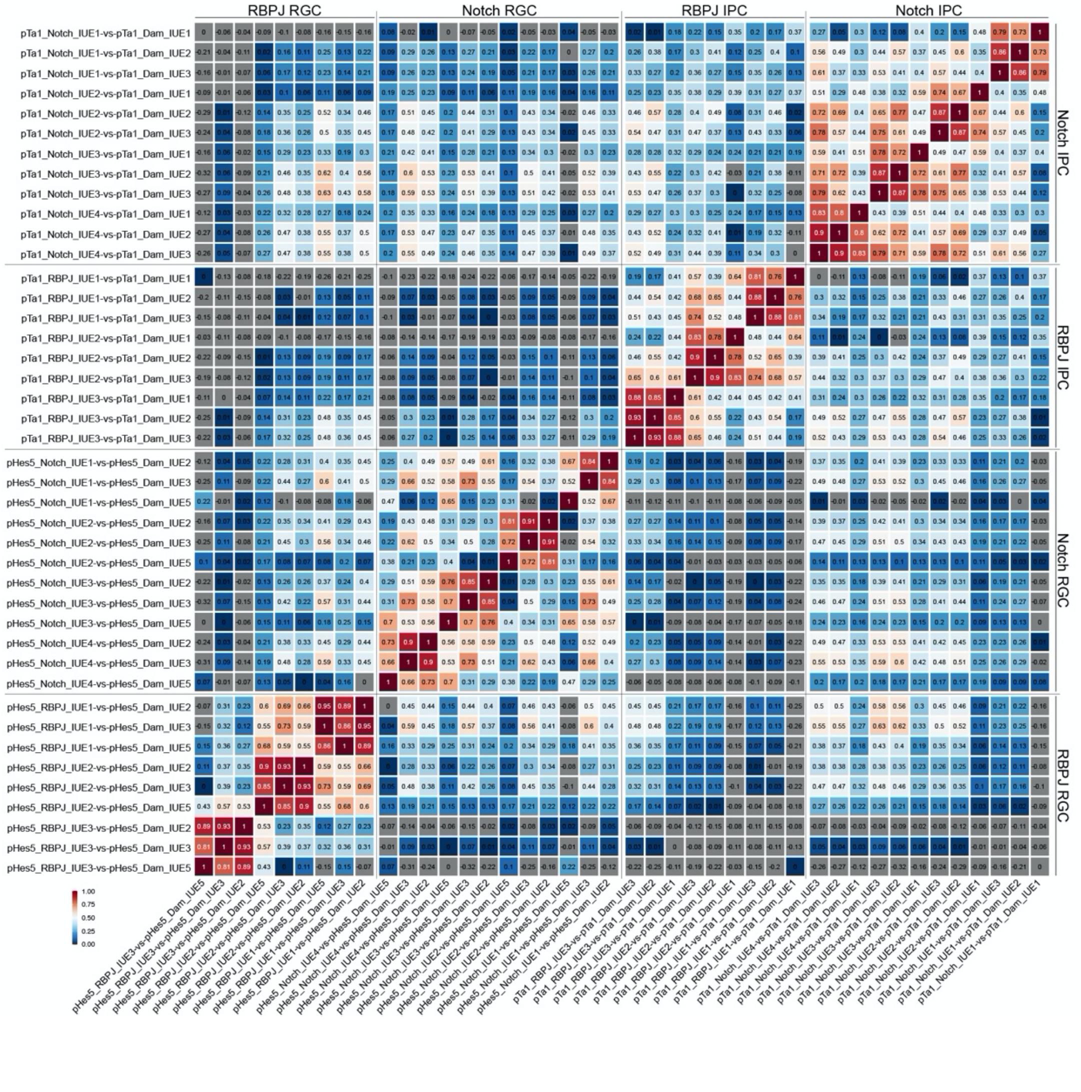
RBPJ RGC
Notch RGC
RBPJ IPC
Notch IPC
pTa1_Notch_IUE1-vs-pTa1_Dam_IUE1
pTa1_Notch_IUE1-vs-pTa1_Dam_IUE2
pTa1_Notch_IUE1-vs-pTa1_Dam_IUE3
pTa1_Notch_IUE2-vs-pTa1_Dam_IUE1
pTa1_Notch_IUE2-vs-pTa1_Dam_IUE2
pTa1_Notch_IUE2-vs-pTa1_Dam_IUE3
pTa1_Notch_IUE3-vs-pTa1_Dam_IUE1
pTa1_Notch_IUE3-vs-pTa1_Dam_IUE2
pTa1_Notch_IUE3-vs-pTa1_Dam_IUE3
pTa1_Notch_IUE4-vs-pTa1_Dam_IUE1
pTa1_Notch_IUE4-vs-pTa1_Dam_IUE2
pTa1_Notch_IUE4-vs-pTa1_Dam_IUE3
pTa1_RBPJ_IUE1-vs-pTa1_Dam_IUE1
pTa1_RBPJ_IUE1-vs-pTa1_Dam_IUE2
pTa1_RBPJ_IUE1-vs-pTa1_Dam_IUE3
pTa1_RBPJ_IUE2-vs-pTa1_Dam_IUE1
pTa1_RBPJ_IUE2-vs-pTa1_Dam_IUE2
pTa1_RBPJ_IUE2-vs-pTa1_Dam_IUE3
pTa1_RBPJ_IUE3-vs-pTa1_Dam_IUE1
pTa1_RBPJ_IUE3-vs-pTa1_Dam_IUE2
pTa1_RBPJ_IUE3-vs-pTa1_Dam_IUE3
pHes5_Notch_IUE1-vs-pHes5_Dam_IUE2
pHes5_Notch_IUE1-vs-pHes5_Dam_IUE3
pHes5_Notch_IUE1-vs-pHes5_Dam_IUE5
pHes5_Notch_IUE2-vs-pHes5_Dam_IUE2
pHes5_Notch_IUE2-vs-pHes5_Dam_IUE3
pHes5_Notch_IUE2-vs-pHes5_Dam_IUE5
pHes5_Notch_IUE3-vs-pHes5_Dam_IUE2
pHes5_Notch_IUE3-vs-pHes5_Dam_IUE3
pHes5_Notch_IUE3-vs-pHes5_Dam_IUE5
pHes5_Notch_IUE4-vs-pHes5_Dam_IUE2
pHes5_Notch_IUE4-vs-pHes5_Dam_IUE3
pHes5_Notch_IUE4-vs-pHes5_Dam_IUE5
pHes5_RBPJ_IUE1-vs-pHes5_Dam_IUE2
pHes5_RBPJ_IUE1-vs-pHes5_Dam_IUE3
pHes5_RBPJ_IUE1-vs-pHes5_Dam_IUE5
pHes5_RBPJ_IUE2-vs-pHes5_Dam_IUE2
pHes5_RBPJ_IUE2-vs-pHes5_Dam_IUE3
pHes5_RBPJ_IUE2-vs-pHes5_Dam_IUE5
pHes5_RBPJ_IUE3-vs-pHes5_Dam_IUE2
pHes5_RBPJ_IUE3-vs-pHes5_Dam_IUE3
pHes5_RBPJ_IUE3-vs-pHes5_Dam_IUE5
Notch IPC
RBPJ IPC
Notch RGC
RBPJ RGC
1.00
0.75
0.50
0.25
0.00

Supplementary Fig. 3. **Genome-wide Pearson correlation matrices**. Pearson correlation coefficients of linear correlations between all replicates across the four experimental conditions (see Fig. 2a) and the Dam-only conditions (see Fig. 5a) based on 500bp bins across the genome (**a**); and between the normalised datasets (see Fig. 2a) based on the peak coordinates identified across the four experimental conditions (**b**).

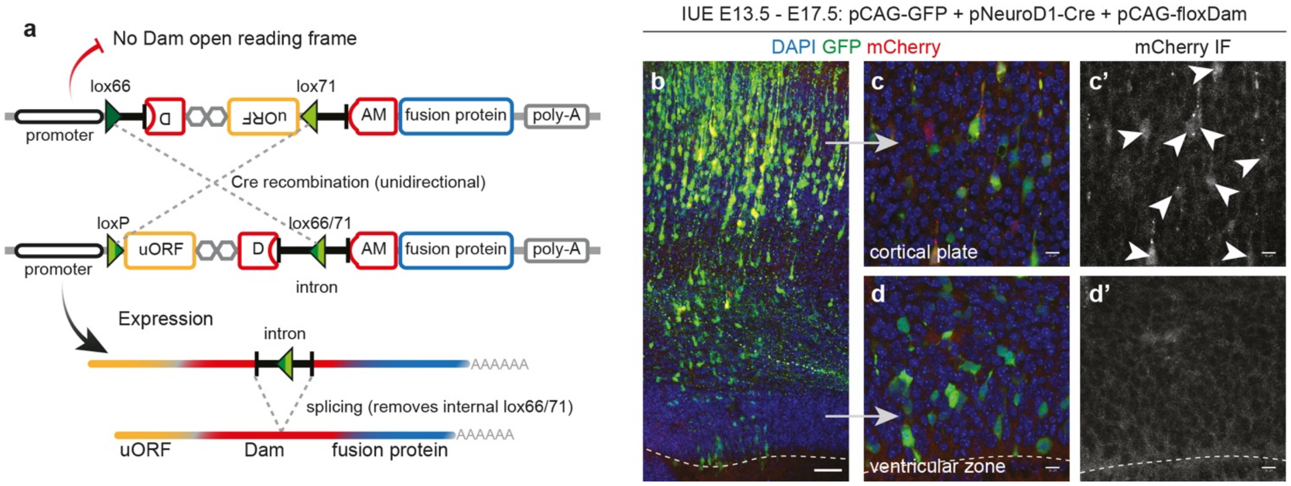

Supplementary Fig. 4. Cre-inducible construct (floxDam). **a**. Unidirectional Cre-induced recombination reconstitutes the Dam coding sequence and the internal Lox66/71-site is removed from the transcript by splicing. **b-d**. *In utero* electroporation of pNeuroD1-Cre with pCAG-floxDam and pCAG-Venus at E13.5; coronal sections at E17.5 stained for DAPI (nuclei, blue), GFP (green) and mCherry (uORF, red). Dashed lines mark the apical side of the VZ. Arrowheads indicate mCherry-expressing cells. Scale bars are 50µm (**b**) and 10µm (**c,d**).

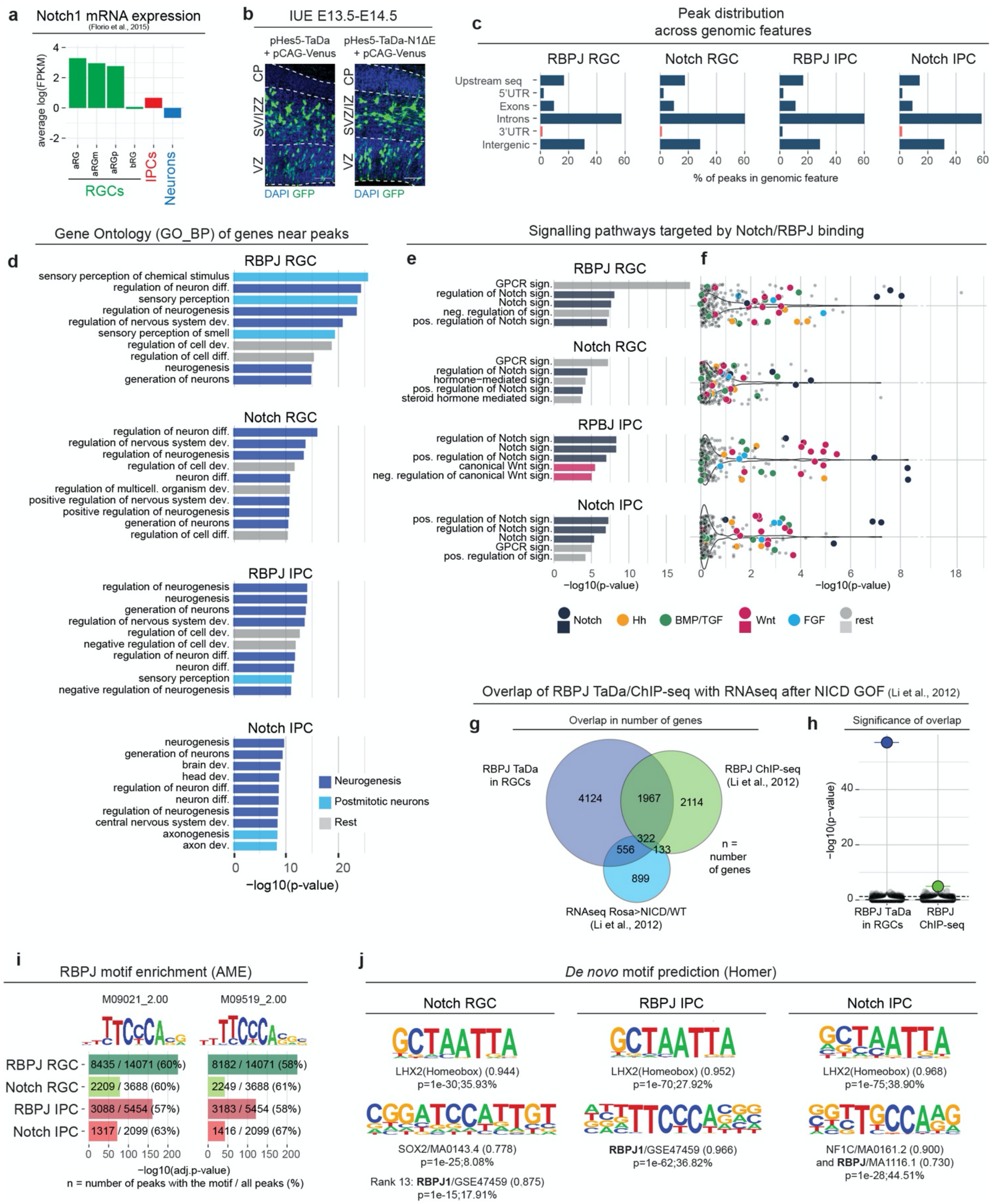

Supplementary Fig. 5. Cell-type specific *in utero* TaDa of Notch and RBPJ. **a.** Notch1 mRNA expression (average log(FPKM) values) in RGCs, IPCs and neurons from bulk RNA-seq [34]. **b**. IUE at E13.5 of pCAG-Venus with Dam or N1ΔE-Dam under control of pHes5, stained at E14.5 for DAPI (nuclei, blue) and GFP (Venus, green). Dashed lines mark the VZ, SVZ/IZ and CP. Scale bars are 50µm. **c**. Relative peak distribution in relation to genomic features. **d-f**. Gene ontology analysis of genes associated with peaks, showing all terms from GO_BP (Biological Processes) (**d**), or terms containing "signalling" (**e,f**). Specific categories or signalling pathways are highlighted in the indicated colours. P-values derive from the enrichment model as implemented in the broadenrich command of the chipenrich package. **g,h**. Overlap of genes differentially expressed in the cortex upon NICD overexpression (RNA-seq [37], light blue) and bound by RBPJ (*in utero* TaDa

in RGCs, green, or RBPJ ChIP-seq [37], dark blue (**g**). Significance values of the observed overlaps compared to expected overlap for these gene numbers and sizes; p-values calculated via hypergeometric test (**h**). **i**. RBPJ motif enrichment; p-values calculated via Fisher's exact test implemented in AME of the MEME suite. **j.** *De novo* motif prediction; p-values calculated via Homer based on a binomial distribution.

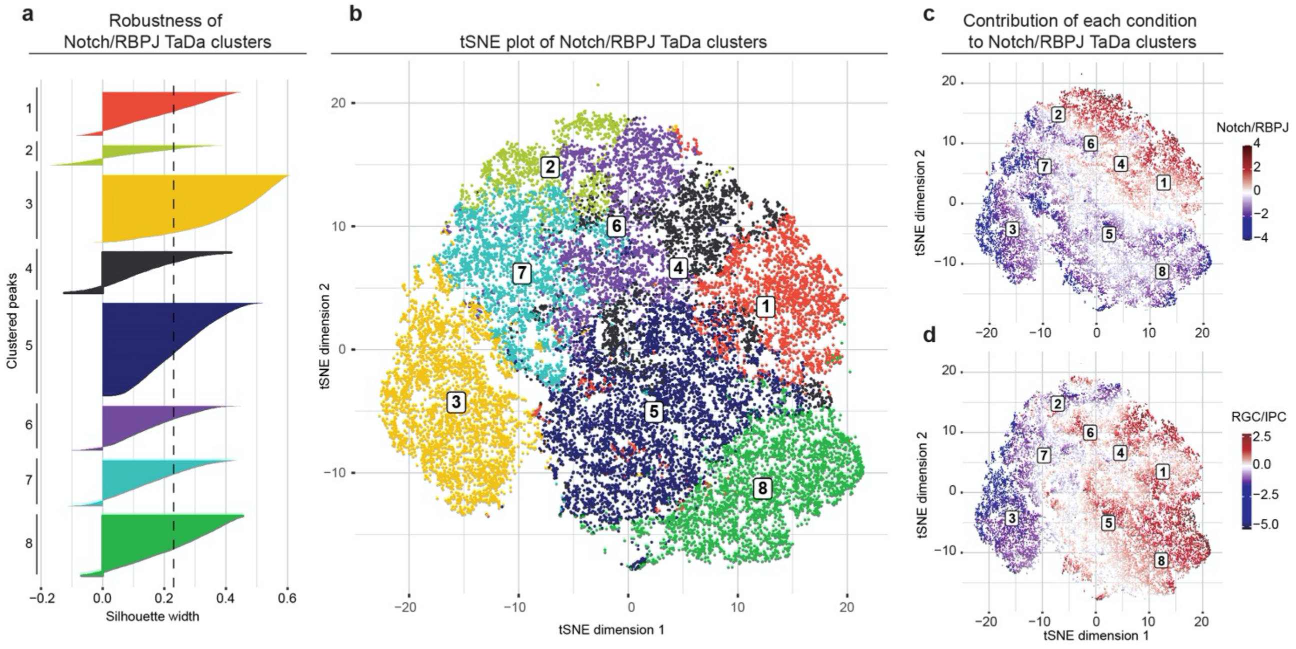

Supplementary Fig. 6. k-means clustering for Notch/RBPJ peak regions. a. Silhouette plot for the clustering. Silhouettes are sorted according to distance of a peak to the centre of the neighbouring cluster which determines order in Fig. 3a. **b**. tSNE plot representing distribution of peaks by dimensionality reduction of aggregated binding intensities of peaks. Colours indicate cluster affiliation. **c,d**. Ratios of summed binding intensities for assayed proteins (Notch/RBPJ) (**c**) and cell types (RGC/IPC) (**d**) overlaid on tSNE evaluates dimension reduction according to these two factors.

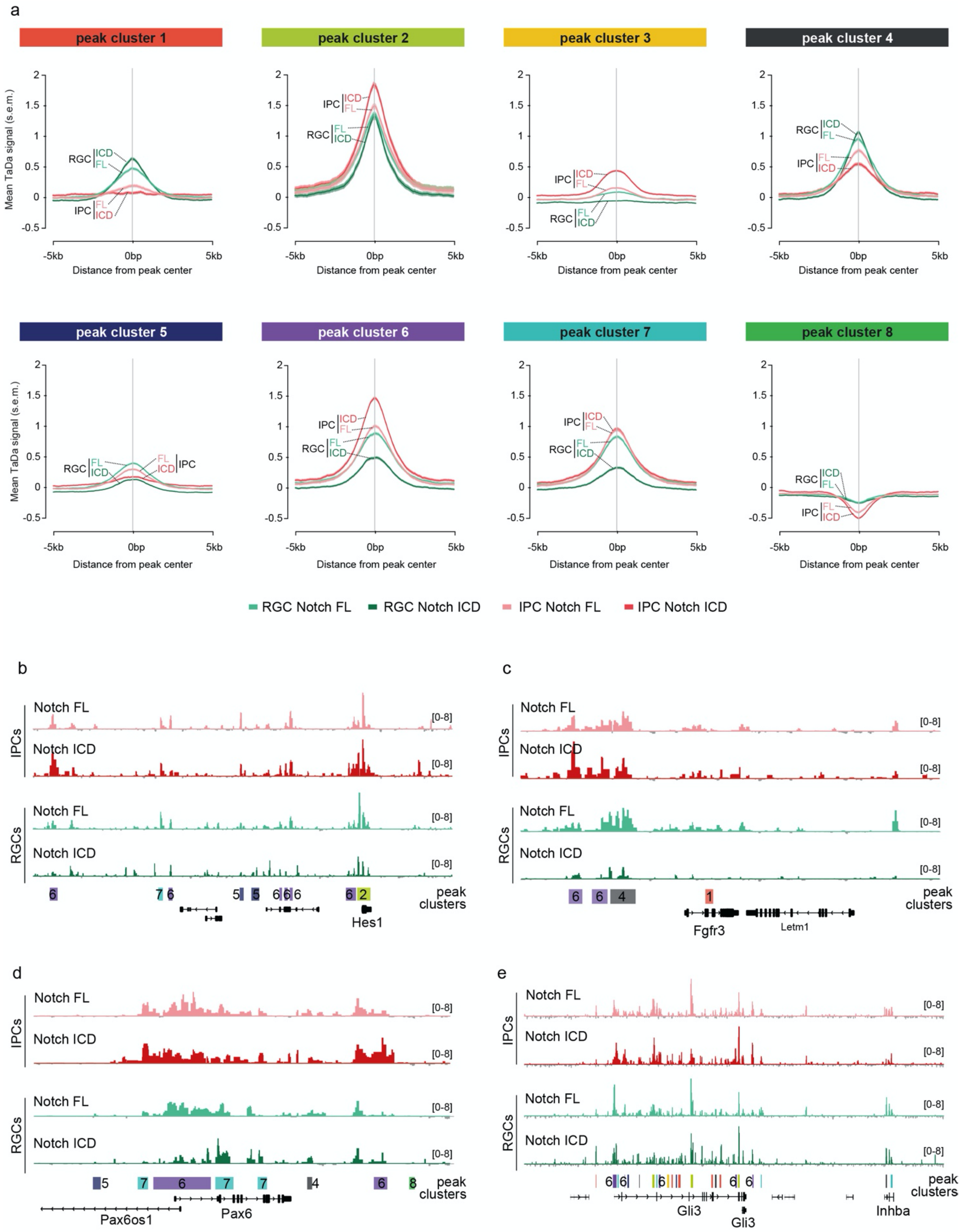

Supplementary Fig. 7. **TaDa in RGCs and IPCs with full-length Notch**. **a**. Average signal intensity (±s.e.m, shaded area) in RGCs (green) and IPCs (red) on peaks from the indicated Notch/RBPJ peak clusters for full-length Notch TaDa (Notch FL) or NICD TaDa (Notch ICD). **b-e**. Binding profiles for TaDa in RGCs or IPCs with full-length (FL) Notch and Notch ICD near RGC-specific Notch target genes *Hes1* (**b**), *Fgfr3* (**c**), *Pax6* (**d**) and *Gli3* (**e**) associated with cluster 6 peaks.

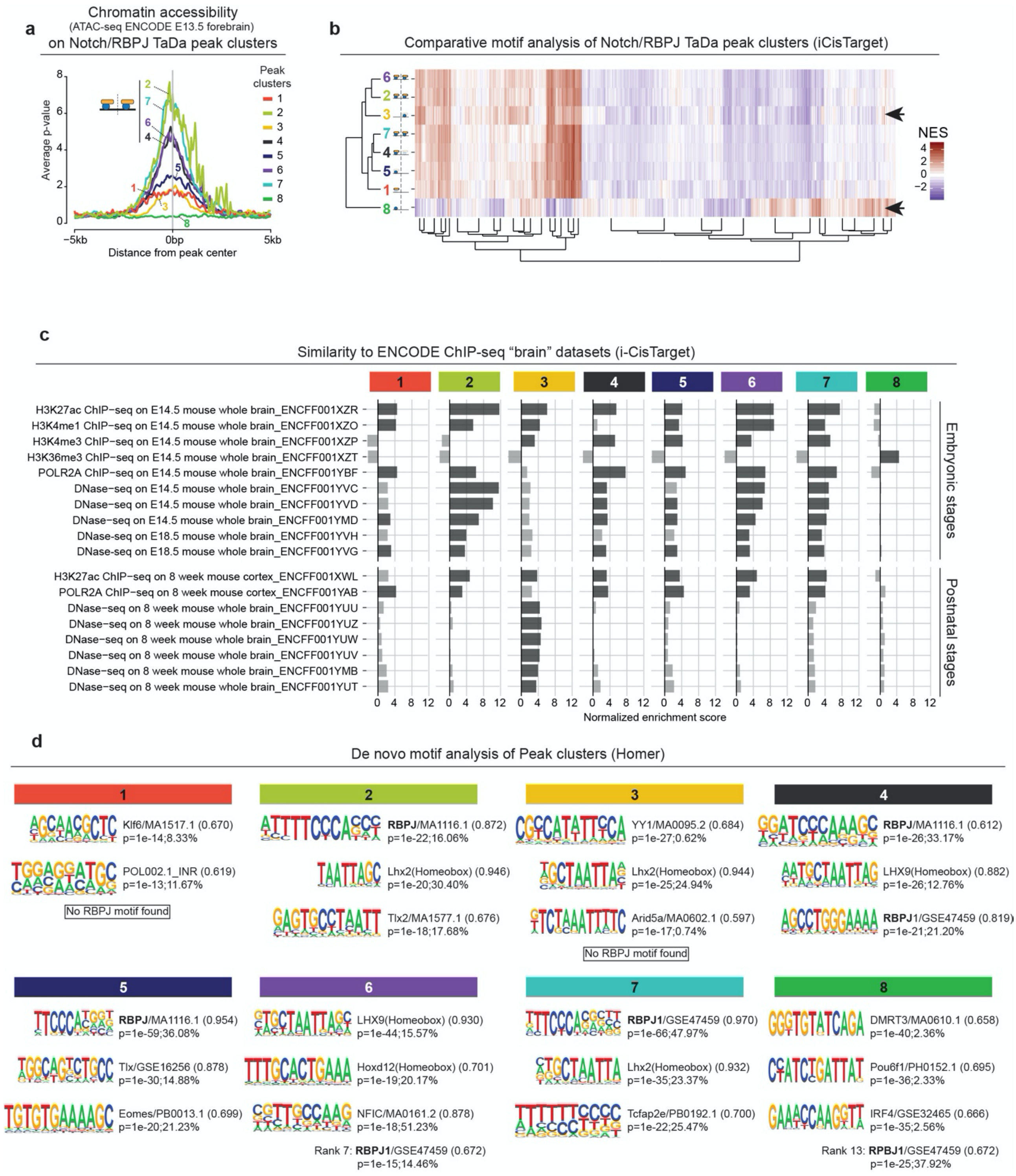

Supplementary Fig. 8. Properties of Notch/RBPJ peak clusters. **a**. Average p-value from E13.5 ATAC-seq (values from ENCODE [31]) plotted on NOTCH/RBPJ peak clusters. **b**. Normalized enrichment scores (NES) and hierarchical clustering of motifs detected by i-CisTarget [47] under peaks of each NOTCH/RBPJ peak cluster. **c**. Similarity in genome-wide distribution between peaks of each cluster and the indicated datasets from ENCODE [31], as determined by i-CisTarget [47]. Dark grey indicates significant similarity. **d**. *De novo* motif analysis of sequences under the peaks of each cluster. P-values calculated via Homer based on a binomial distribution. Top 3 position weight matrices are shown, with the name of the best matching motif and its similarity score, p-value of the motif and percentage of peaks where the motif is detected.

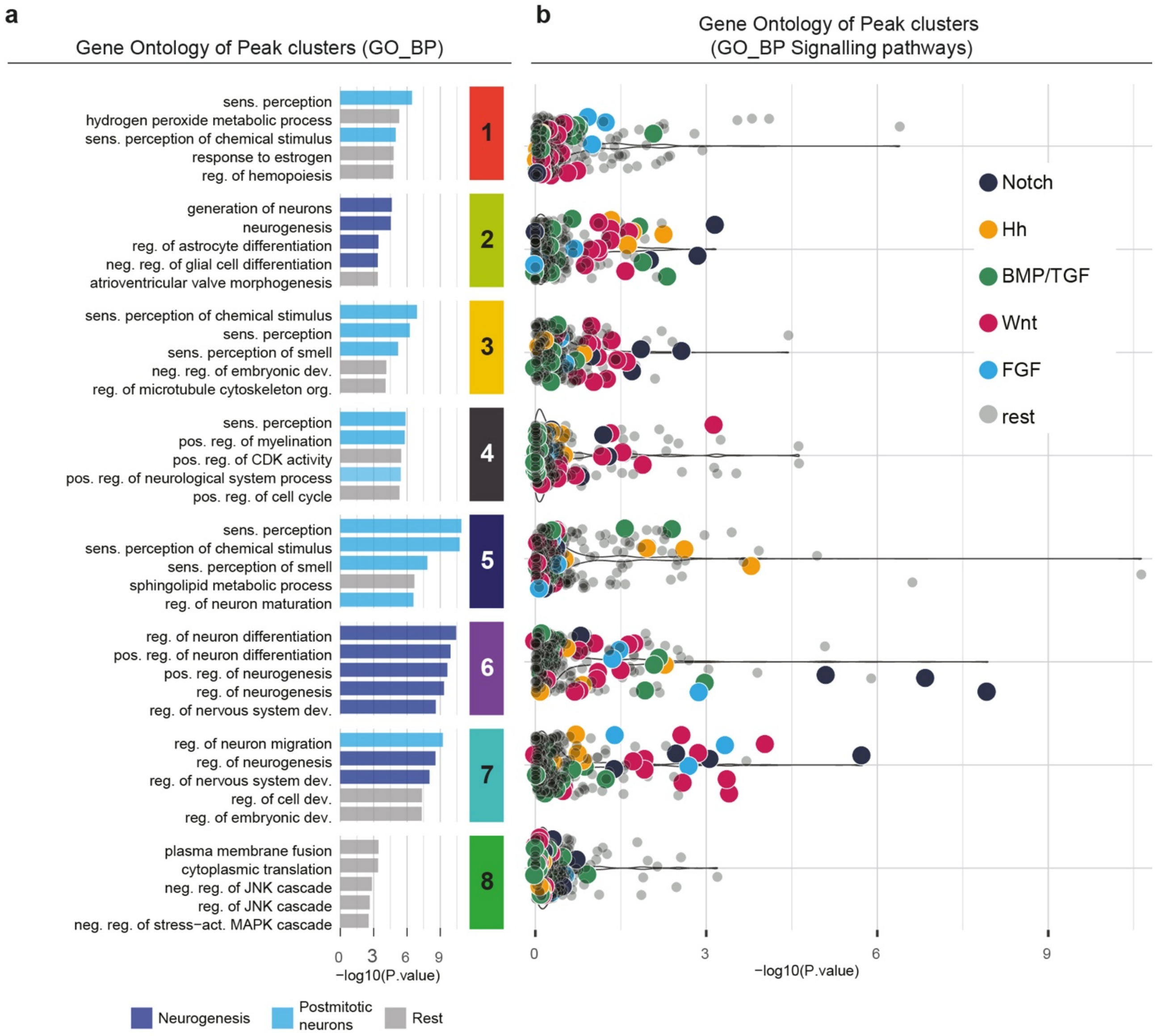

Supplementary Fig. 9. Gene ontology analysis of NOTCH/RBPJ-bound clusters. a,b. Gene ontology analysis of genes associated with peaks in each Notch/RBPJ peak cluster, showing all terms from GO_BP (Biological Processes) (**a**), or terms containing "signalling" (**b**). Specific categories or signalling pathways are highlighted in the indicated colours. P-values derive from the enrichment model as implemented in the broadenrich command of the chipenrich package.

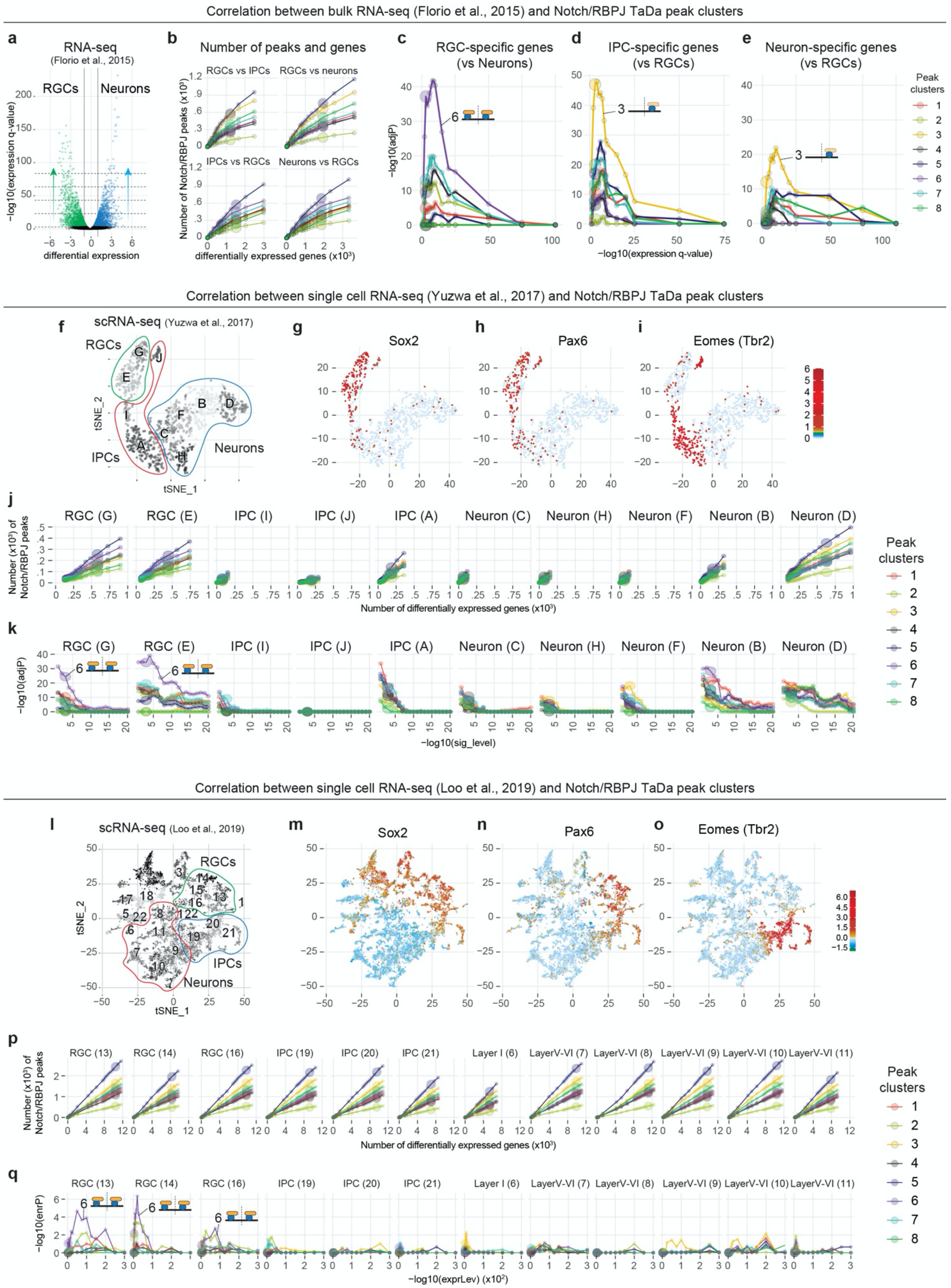

Supplementary Fig. 10. **Genome-wide comparison of cell-type specific expression data and Notch/RBPJ binding patterns**. **a**. Genes differentially expressed in RGCs compared to neurons from bulk RNA-seq. **b**. Number of genes differentially expressed in RGCs, IPCs or neurons (x-axis) at decreasing q-values (datapoints; q=0.05 is enlarged), and numbers of peaks from each peak cluster (y-axis) associated with the differentially expressed genes. **c-e**. Enrichment of Notch/RBPJ

peak clusters near genes differentially expressed at different q-values in RGCs compared to neurons (**c**), IPCs compared to RGCs (**d**) and neurons compared to RGCs (**e**). **f-q**. tSNE-plot of single-cell RNA-seq from Yuzwa et al. [35] (**f-i**) or Loo et al. [50] (**l-o**) indicating the different scSeq clusters and the cell-type annotations (**f,l**), or the expression level of the indicated marker genes (**g-i,m-o**). **j,p**. Number of genes differentially expressed in the cells from the indicated scSeq cluster compared to all other cells (x-axis) at decreasing q-values (datapoints; q=0.05 is enlarged) and the number of peaks from each peak cluster (y-axis) associated with the differentially expressed genes. Note that some scSeq clusters from Yuzwa et al (**j**) (I,J,C,H,F) have very few genes specifically expressed in their cells. **k,q**. Enrichment of Notch/RBPJ peak clusters near genes differentially expressed in the indicated scSeq cluster at different q-values.

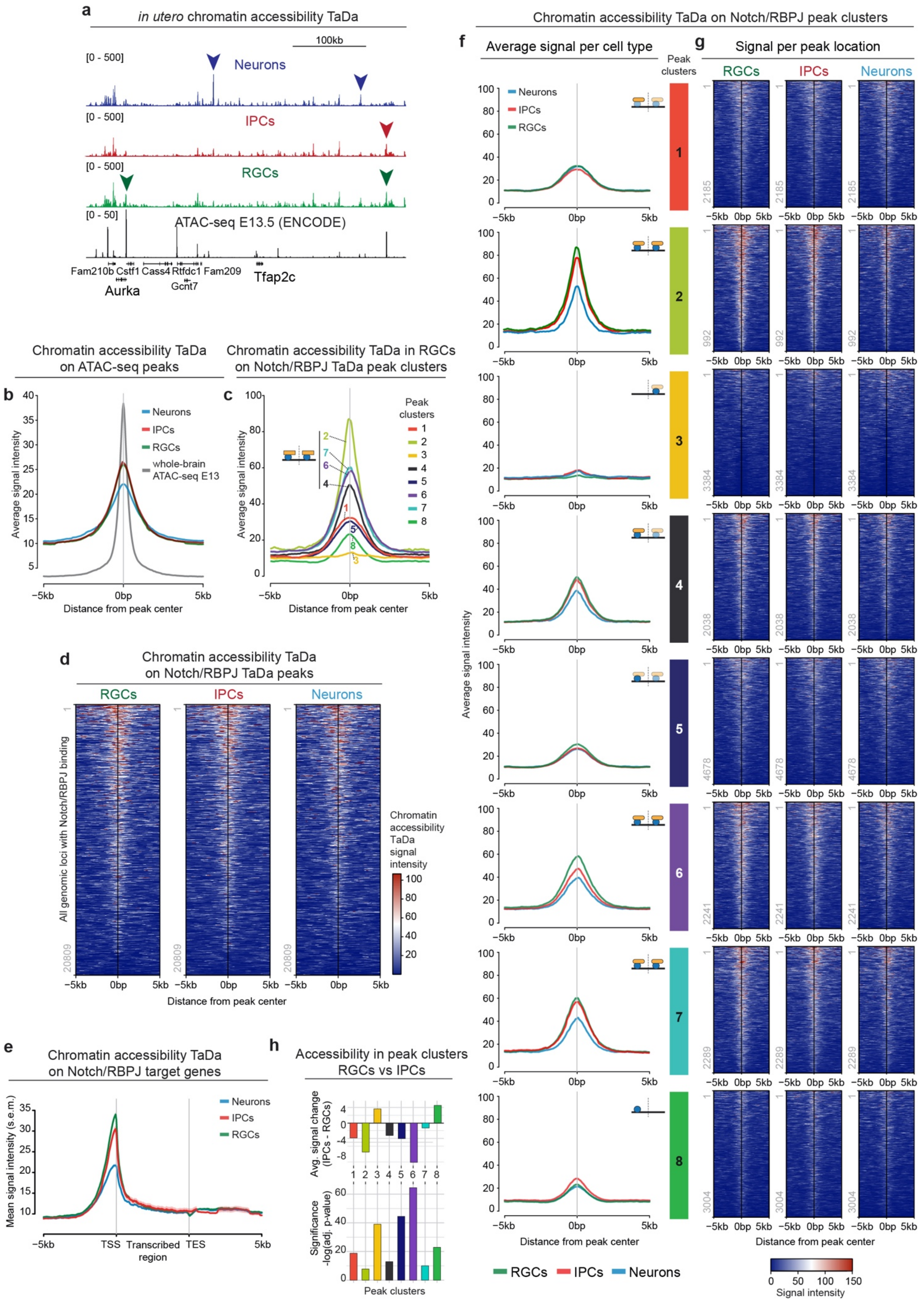

Supplementary Fig. 11. Cell-type specific *in utero* chromatin accessibility TaDa. **a**. I*n utero* chromatin accessibility profiles in RGCs, IPCs and post-mitotic neurons and of E13.5 forebrain ATAC-seq (ENCODE) at the *Tfap2c* locus. Arrowheads indicate cell-type specific differences in

signal intensity. **b-g**. Average signal intensity (**b,c,e,f**) or signal intensity (**d,g**) in RGCs (**c**) or in RGCs, IPCs and neurons (**b,d-g**) on ATAC-seq peaks (FDR<10^{-2}) (**b**), on peaks from the indicated Notch/RBPJ peak clusters (**c,f,g**), on all genomic loci with NOTCH or RBPJ binding (**d**), or across all Notch/RBPJ target genes and the 5kb upstream and downstream genomic region (**e**). **h**. Change in average signal (bottom) and its associated adjusted p-value (top) of chromatin accessibility TaDa from RGCs to IPCs (compare to signal in (**f**)) on the indicated peak clusters. P-values calculated via Wilcoxon rank sum test with Bonferroni correction for multiple testing.
