# Supplementary material for: Reduced chromatin accessibility correlates with resistance to Notch activation
Source: Nat Commun. 2022 Apr 25;13:2210. doi: 10.1038/s41467-022-29834-z (PMC9039071; doi:10.1038/s41467-022-29834-z)
Supplement: Supplementary file 2 — Description of additional Supplementary File [file 41467_2022_29834_MOESM2_ESM.pdf]

Descriptions of Additional Supplementary Data files

Supplementary Data 1. Loci bound by NOTCH/RBPJ in RGCs or IPCs and their associated genes. Related to Fig. 2.

Supplementary Data 2. Overlap of genes bound by RBPJ ChIP-seq, RGC-specific RBPJ TaDa and RNAseq upon NICD overexpression. Related to Fig. S5g.

Supplementary Data 3. Peaks associated with each Notch/RBPJ peak cluster and the associated gene for each peak. Related to Fig. 3.

Supplementary Data 4. RGC-specific genes determined by bulk RNA-seq that are bound by NOTCH/RBPJ in peak cluster 6. Related to Fig. 4b
