# Supplementary material for: Reduced chromatin accessibility correlates with resistance to Notch activation
Source: Nat Commun. 2022 Apr 25;13:2210. doi: 10.1038/s41467-022-29834-z (PMC9039071; doi:10.1038/s41467-022-29834-z)
Supplement: Supplementary file 7 — Reporting Summary [file 41467_2022_29834_MOESM7_ESM.pdf]

nature research

Corresponding author(s): Andrea H. Brand

Last updated by author(s): Nov 24, 2021

Reporting Summary

Nature Research wishes to improve the reproducibility of the work that we publish. This form provides structure for consistency and transparency in reporting. For further information on Nature Research policies, see our Editorial Policies and the Editorial Policy Checklist.

Statistics

For all statistical analyses, confirm that the following items are present in the figure legend, table legend, main text, or Methods section.

n/a	Confirmed	
☐	☒	The exact sample size (*n*) for each experimental group/condition, given as a discrete number and unit of measurement
☐	☒	A statement on whether measurements were taken from distinct samples or whether the same sample was measured repeatedly
☐	☒	The statistical test(s) used AND whether they are one- or two-sided<br>*Only common tests should be described solely by name; describe more complex techniques in the Methods section.*
☒	☐	A description of all covariates tested
☐	☒	A description of any assumptions or corrections, such as tests of normality and adjustment for multiple comparisons
☐	☒	A full description of the statistical parameters including central tendency (e.g. means) or other basic estimates (e.g. regression coefficient) AND variation (e.g. standard deviation) or associated estimates of uncertainty (e.g. confidence intervals)
☐	☒	For null hypothesis testing, the test statistic (e.g. *F*, *t*, *r*) with confidence intervals, effect sizes, degrees of freedom and *P* value noted<br>*Give P values as exact values whenever suitable.*
☒	☐	For Bayesian analysis, information on the choice of priors and Markov chain Monte Carlo settings
☐	☒	For hierarchical and complex designs, identification of the appropriate level for tests and full reporting of outcomes
☐	☒	Estimates of effect sizes (e.g. Cohen's *d*, Pearson's *r*), indicating how they were calculated

Our web collection on statistics for biologists contains articles on many of the points above.

Software and code

Policy information about availability of computer code

Data collection	Sequencing data was acquired on a HiSeq1500 with corresponding Illumina sequencing software. FastQC (v0.11.5) was used to ensure the quality of the acquired *.fastq-files, TrimGalore (v0.4.5) to trim the reads and the Clumpify tool of the BBmap suite for deduplicating reads if necessary (v38.12; dedupe subs=0).
Data analysis	Bowtie2 (v2.3.4.2) allowed read mapping, damidseq_pipeline and accessory scripts (v1.4.5) were used for integrated normalization and averaging, bedGraphToBigWig (v4) for file conversion and IGV (v2.4.19) for data visualization. Macs2 (v2.1.2) enabled peak calling, biomaRt (v2.38.0) acquisition of gene and genome annotations, bedr (v1.0.7), bedtools (v2.26.0) and gat (v1.3.2) associating peaks to genes. Average associations and heatmaps were generated with seqplots (v1.12.1). The R packages factoextra (v1.0.5), clValid (v0.6-6), mclust (v5.4.5) and cluster (v2.0.7-1) facilitated clustering, Rtsne (v0.15) dimensionality reduction and rtracklayer (v1.42.2) for mm10-to-mm9-lift of coordinates. Homer (v4.10), the MEME-suite (v5.0) and i-cisTarget (v5.0) provided online platforms for integrated motif analysis. The R packages stats (v3.6.1), ggdendro (v0.1.20) and dendextend (v1.10.0) were used for hierachical clustering, Biostrings (v2.50.2) for acquiring sequences corresponding to peak regions, chipenrich (v2.10.0) for GO-term enrichment, AnnotationDbi (v1.48.0) and org.Mm.eg.db (v3.10.0) for converting gene identifiers. BamCoverage of the deepTools suite (v3.1.3) permitted read extension and the R stats package (v3.6.1) subsequently calculation of Spearman Correlation Coefficients.<br>Publicly available RNAseq-data was psuedoaligned with kallisto (v0.45.0) and analysed with sleuth (v.0.30.0) and corresponding scRNAseq-data with the R packages methods (v3.6.1) and Seurat (v2.3.4/v3.0.2). Statistical modelling and p-value adjustment was performed with the R stats package (v3.6.1).

For manuscripts utilizing custom algorithms or software that are central to the research but not yet described in published literature, software must be made available to editors and reviewers. We strongly encourage code deposition in a community repository (e.g. GitHub). See the Nature Research guidelines for submitting code & software for further information.

Data

Policy information about availability of data

All manuscripts must include a data availability statement. This statement should provide the following information, where applicable:

- Accession codes, unique identifiers, or web links for publicly available datasets
- A list of figures that have associated raw data
- A description of any restrictions on data availability

DamID sequencing data acquired as part of this study, covering all analysis and supporting results throughout this study were made accessible under the accession code GSE152207 in NCBI's Gene Expression Omnibus (https://www.ncbi.nlm.nih.gov/geo/query/acc.cgi?acc=GSE152207).
Single-cell RNAseq data accompanying 'Yuzwa, SA et al., 2017' and 'Loo, L et al., 2019' were obtained from GEO under the accession numbers GSE107122 and GSE123335. The bulk RNAseq dataset published in 'Florio, M et al., 2015' was accessed via the corresponding accession number GSE65000.
E13.5 mouse brain ATACseq data was retrieved from the ENCODE database (i.e., ENCFF450ZSN.bigWig, ENCFF798QON.bam).
While the raw data for RBPJ ChIPseq and RNAseq after NICD overexpression in the mouse cortex as presented in 'Li, Y et al., 2012' is not available anymore, we used the peak information provided in Supplementary Table 5 and the information on differentially expressed genes provided in Supplementary Table 1 attached to the corresponding publication (https://stemcellsjournals.onlinelibrary.wiley.com/doi/10.1002/stem.1030).

Field-specific reporting

Please select the one below that is the best fit for your research. If you are not sure, read the appropriate sections before making your selection.

☒ Life sciences ☐ Behavioural & social sciences ☐ Ecological, evolutionary & environmental sciences

For a reference copy of the document with all sections, see nature.com/documents/nr-reporting-summary-flat.pdf

Life sciences study design

All studies must disclose on these points even when the disclosure is negative.

Sample size	The number of samples was established as a trade off between ensuring reproducibility and minimizing the amount of animal experiments. Sample-size and power calculations were not performed. To ensure reproducibility we collected at least 3 replicates for each genotype and condition as is standard in the field: 3-7 replicates were acquired for Dam-Only samples in their respective cell types (i.e., 3 neurons, 4 intermediate progenitors, 5 radial glial cells, 7 ubiquitous expression). 3 RBPJ-Dam and 4 Notch-Dam replicates were obtained for each, intermediate progenitors and radial glial cells. 4 full-length Notch-Dam replicates were generated for radial glial cells and 2 for intermediate progenitors. For full-length Notch-Dam in intermediate progenitors, only 2 samples were available, due to time restrictions during the revision process.
Data exclusions	Minor variations during the in-utero electroporation procedure can lead to insufficient expression from the plasmid. Calculating genome-wide signal-to-noise ratios and Spearman correlation coefficients allowed to detect outliers, which were excluded from any further analysis.
Replication	Correlations between replicates were assessed to ensure genome-wide reproducibility amongst acquired replicates for each plasmid-celltype combination and results are presented in Supplemental Figure 3.
Randomization	Experimental groups were assigned according to the particular plasmids electoroporated, which determine the celltype of expression and the specific DamID construct. Any additional grouping was derived analytically based on the data itself (i.e., peak clusters). No randomization was conducted.
Blinding	None of the investigators were blinded during assignment of groups for sequencing-based experiments or data analysis. Blinding was not relevant, since all sequencing data were acquired by sequencing rather than subjective measurements. Batch effects indicating potential covariates or variation during data acquisition were accounted for by normalization. Acquisition of cell counts for Fig. 1j could not be performed in a blinded manner as the phenotype was too apparent compared to the control samples. Cell counts for Supplementary Fig. 2d in turn were obtained in a blinded manner.

Reporting for specific materials, systems and methods

We require information from authors about some types of materials, experimental systems and methods used in many studies. Here, indicate whether each material, system or method listed is relevant to your study. If you are not sure if a list item applies to your research, read the appropriate section before selecting a response.

Materials & experimental systems

n/a	Involved in the study
	[x] Antibodies
[x]	Eukaryotic cell lines
[x]	Palaeontology and archaeology
	[x] Animals and other organisms
[x]	Human research participants
[x]	Clinical data
[x]	Dual use research of concern

Methods

n/a	Involved in the study
[x]	ChIP-seq
[x]	Flow cytometry
[x]	MRI-based neuroimaging

Antibodies

Antibodies used	chicken anti-GFP (Abcam ab13970), 1:1000 dilution<br>rabbit anti-RFP (Abcam ab62341), 1:500 dilution<br>goat anti-Sox2 (R&D AF2018), 1:500 dilution<br>rabbit anti-Sox9 (Millipore AB5535), 1:500 dilution<br>rabbit anti-Prdm16 (gift from P. Seale), 1:200 dilution<br>rabbit anti-Pax6 (Covance PRB-278P), 1:500 dilution<br>rabbit anti-Hes1 (Cell Signalling D6P2U), 1:100 dilution<br>goat anti-Gli3 (R&D AF3690), 1:500 dilution
Validation	chicken anti-GFP (Abcam ab13970): Antibody was used in at least 2036 publications, including studies involving mice samples and was tested by the manufacturers for immunohistochemistry.<br>rabbit anti-RFP (Abcam ab62341): Antibody was used in at least 184 publications, including studies involving mice specimens and was validated by the manufacturers for immunohistochemistry.<br>goat anti-Sox2 (R&D AF2018): Antibody was used in at least 184 publications, including studies involving mice samples and was tested by the manufacturers for immunohistochemistry.<br>rabbit anti-Sox9 (Millipore AB5535): Antibody was used in at least 155 publications, including studies involving mice samples and was tested by the manufacturers for immunohistochemistry.<br>rabbit anti-Prdm16 (gift from P. Seale): Antibody was used in Seale, P. et al., 2008 (doi:10.1038/nature07182), Seale, P. et al., 2011 (doi:10.1172/JCI44271) and Harms, M.J. et al., 2015 for work in mice.<br>rabbit anti-Pax6 (Covance PRB-278P): Antibody was used in at least 169 publications, including studies involving mice samples and was amongst others used for immunohistochemistry in these studies.<br>rabbit anti-Hes1 (Cell Signalling D6P2U): Antibody was used in at least 72 publications, including studies involving mice samples and was tested by the manufacturers for immunohistochemistry.<br>goat anti-Gli3 (R&D AF3690): Antibody was used in at least 33 publications, including studies involving mice samples and was tested by the manufacturers for immunohistochemistry.

Animals and other organisms

Policy information about studies involving animals; ARRIVE guidelines recommended for reporting animal research

Laboratory animals	All experiments were carried out in wild-type, inbred MF-1 mice. Mice are housed in individually ventilated cages (IVCs) within a bio-barriered facility and are kept in (1.) temperatures between 20-24oC, (2.) 45–65% relative humidity and (3.) 12 h light cycles (light: 7:00 am to 7:00 pm, dark: 7:00 pm to 7:00 am). 'Dawn to dust' - lights come up gradually from 6:30 am and go down gradually from 6:30 pm.
Wild animals	The study did not include any wild animals.
Field-collected samples	The study did not include any specimens, animals or samples collected from the field.
Ethics oversight	Mouse experiments were performed under the Animal (Scientific Procedures) Act 1986 Amendment Regulations 2012 following ethical review by the University of Cambridge Animal Welfare and Ethical Review Body (AWERB).

Note that full information on the approval of the study protocol must also be provided in the manuscript.
